# Supplementary material for: Characterization of full-length p53 aggregates and their kinetics of formation
Source: Biophys J. 2022 Oct 13;121(22):4280–98. doi: 10.1016/j.bpj.2022.10.013 (PMC9703098; doi:10.1016/j.bpj.2022.10.013)
Supplement: Document S1. Figures S1–S10, Tables S1, and S2 [file mmc1.pdf]

Biophysical Journal, Volume *121*

Supplemental information

Characterization of full-length p53 aggregates and their kinetics of formation

Linda Julian, Jason C. Sang, Yunzhao Wu, Georg Meisl, Jack H. Brelstaff, Alyssa Miller, Matthew R. Cheetham, Michele Vendruscolo, Tuomas P.J. Knowles, Francesco Simone Ruggeri, Clare Bryant, Susana Ros, Kevin M. Brindle, and David Klenerman

SUPPLEMENTARY INFORMATION

Characterization of full-length p53 aggregates and their kinetics of formation

Linda Julian[1,2], Jason C. Sang[2], Yunzhao Wu[2], Georg Meisl[2], Jack H. Brelstaff[3,4], Alyssa Miller[5], Matthew R. Cheetham[2], Michele Vendruscolo[5], Tuomas P. J. Knowles[5], Francesco Simone Ruggeri[5,6,7], Clare Bryant[4,8], Susana Ros[1], Kevin M. Brindle[1*], David Klenerman[2,9*]

Affiliations

1. Cancer Research UK Cambridge Institute, University of Cambridge, Cambridge, UK
2. Yusuf Hamied Department of Chemistry, University of Cambridge, Cambridge, UK
3. Department of Clinical Neurosciences, University of Cambridge, Cambridge, UK
4. Department of Medicine, University of Cambridge, Cambridge, UK
5. Centre for Misfolding Diseases, Yusuf Hamied Department of Chemistry, University of Cambridge, Cambridge, UK
6. Present Address: Laboratory of Organic Chemistry, Wageningen University & Research, Wageningen, Netherlands
7. Present Address: Laboratory of Physical Chemistry, Wageningen University & Research, Wageningen, Netherlands
8. Department of Veterinary Medicine, University of Cambridge, Cambridge, UK
9. UK Dementia Research Institute, University of Cambridge, Cambridge, UK

* Corresponding authors

Supplementary Figures

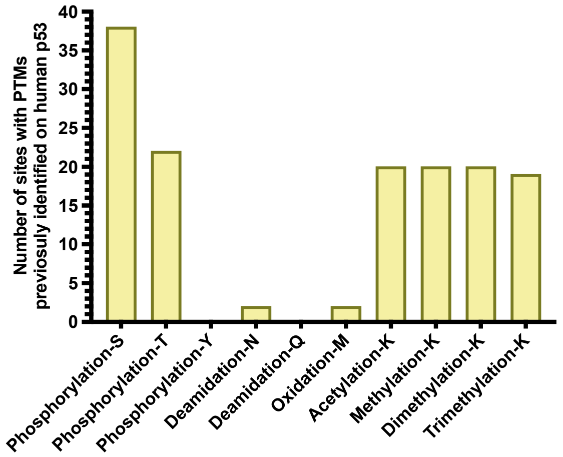

Figure S1. Post-translational modifications reported in human p53. Number of sites with post-translational modifications reported previously in human p53. The modifications include: phosphorylation of serine (S), threonine (T) and tyrosine (Y), deamidation of asparagine (N) and glutamine (Q), oxidation of methionine (M), acetylation and methylation of lysine (K).

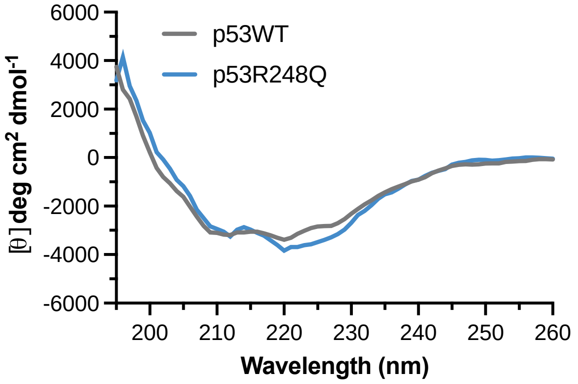

Figure S2. Circular dichroic (CD) spectra of p53WT and p53R248Q. CD spectra were recorded for insect-derived full-length p53WT and p53R248Q at a concentration of 2.5 µM and presented as a plot of molar ellipticity [θ] as a function of wavelength. Values were averaged from three accumulations of spectral scans, baseline corrected and the net spectrum was smoothed using the Savitsky-Golay method.

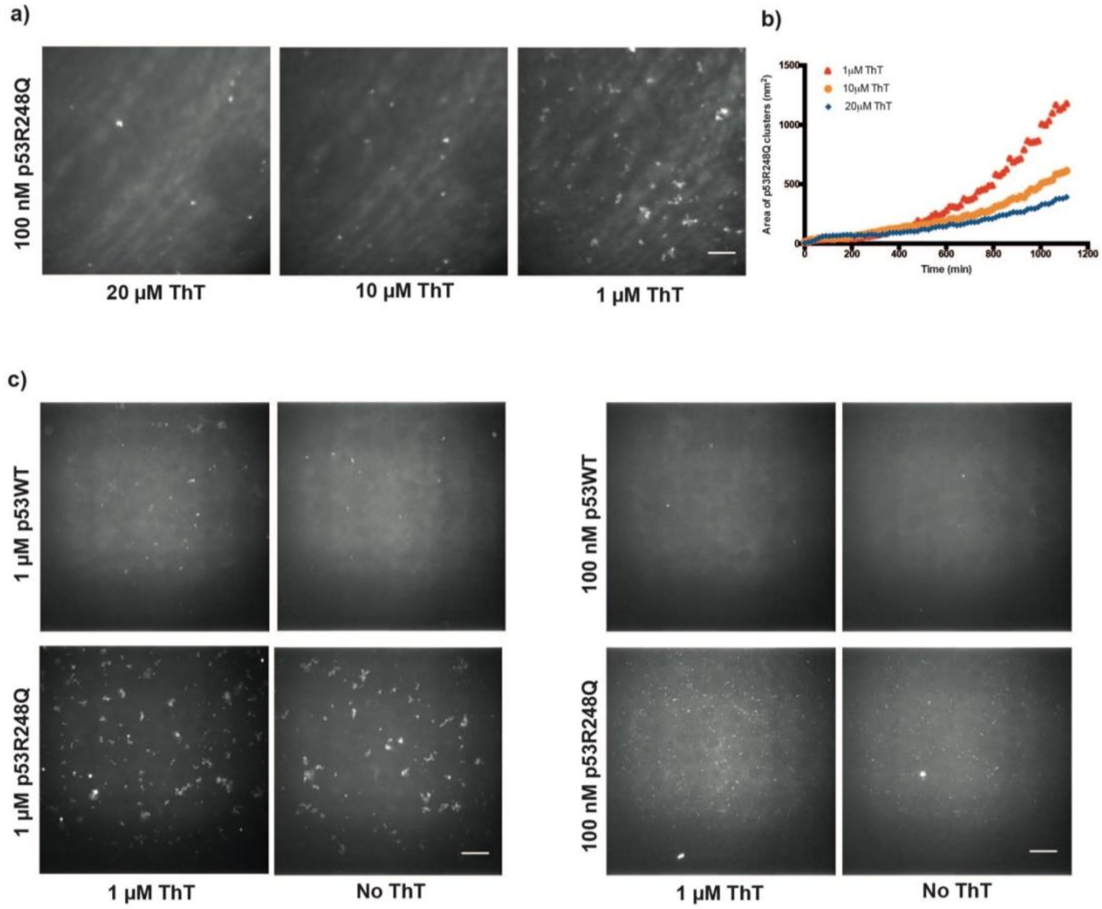

Figure S3. Effect of Thioflavin T (ThT) on p53 aggregation. (**a**) Representative images of insect-derived p53R248 aggregated for 18 hours with different concentrations of ThT. (**b**) Area occupied by p53R248 aggregate clusters as a function of time showing inhibitory effect of high concentration of ThT on aggregation. All experiments were performed in chambered glass slides at 37°C, pH 7.2. (**c**) Representative images of 1 µM and 100 nM p53WT and p53R248Q aggregated for 72 hours in the absence or presence of 1 µM ThT. Scale bar = 10 µm.

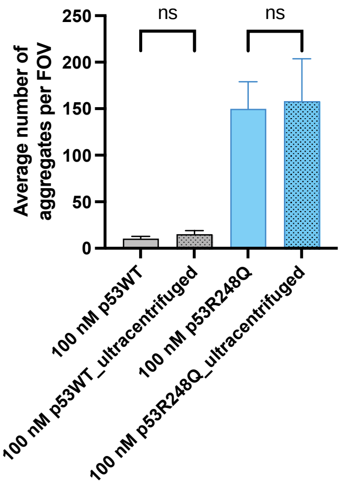

Figure S4. Absence of preformed seeds. Average number of aggregates per field of view (FOV) formed after 72 hours of aggregation of 100 nM p53WT and p53R248Q with and without prior ultracentrifugation. Error bars represents standard error of the mean (ns = not significant).

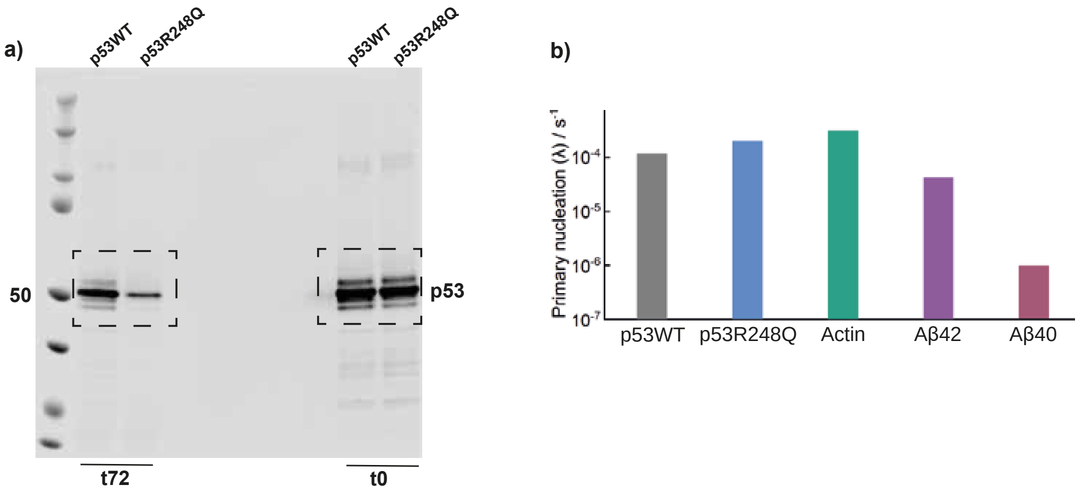

Figure S5. Modelling of p53 aggregation kinetics. (a) Uncropped western blot panels for Figure 3a, boxes with dashed border indicating regions used for the composite**.** (**b**) Bar graph showing primary nucleation rates of p53WT and p53R248Q compared to other aggregating proteins associated with neurodegenerative diseases using published rate constants and evaluated at a monomer concentration of 1 μM.

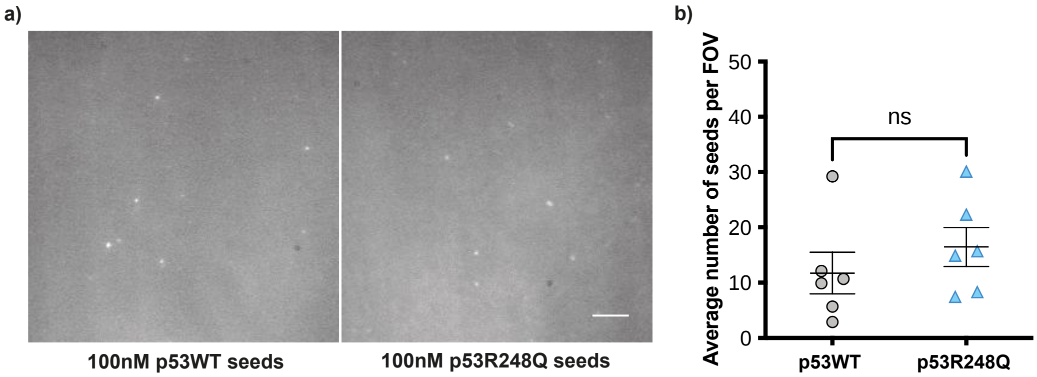

Figure S6. Generation of seeds. (**a**) Representative images showing presence of seeds on glass surface generated from aggregating 100 nM p53WT and p53R248Q for 8 hrs followed by repeated washes to remove remaining monomer. Scale bar = 10 µm. (**b**) Dot plot of average number of p53WT and p53R248Q seeds used for seeded aggregation reactions from 9 fields of view (FOV). Error bars represents standard error of the mean (ns = not significant).

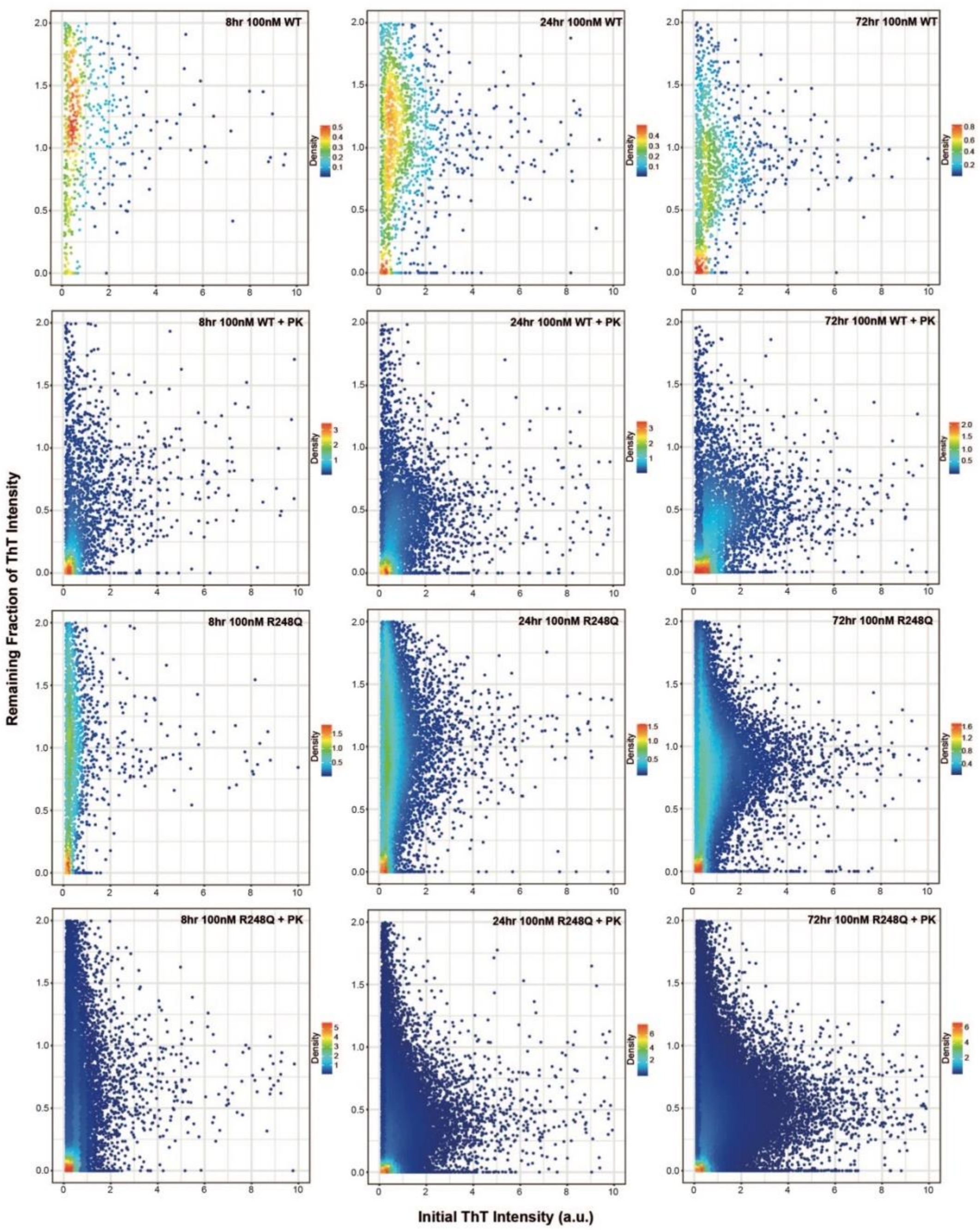

Figure S7. Sensitivity of p53 aggregates to Proteinase K (PK). Density scatter plots showing initial ThT intensity of aggregates of 100 nM p53WT and p53R248Q from different time points of aggregation (8, 24 and 72 hours) and the remaining fraction of ThT intensity after 30 minutes with or without proteolytic digestion.

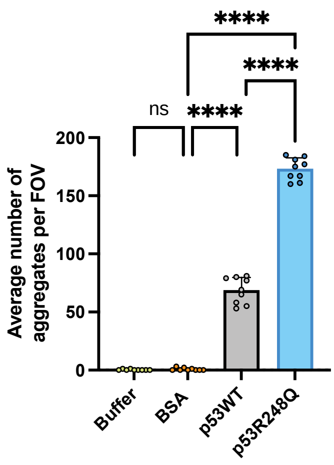

Figure S8. Number of aggregates added to the lipid bilayer. Average number of aggregates per field of view (FOV) formed after 72 hours of aggregation of equivalent concentrations of BSA, p53WT and p53R248Q. Error bars represents standard deviation (ns = not significant, **** P<0.0001).

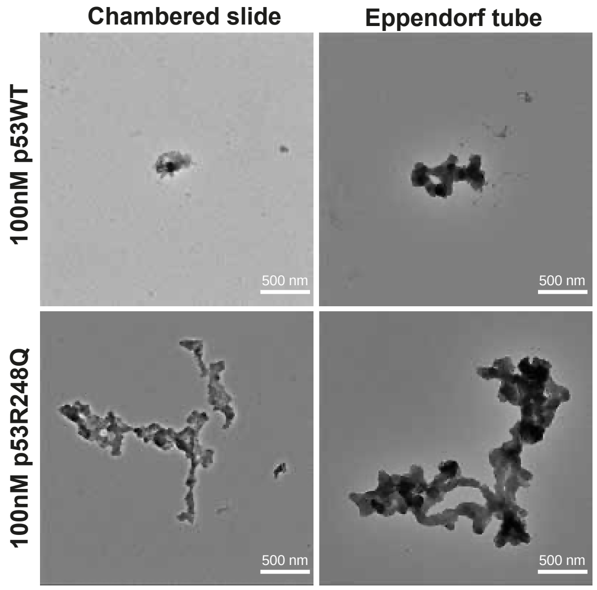

Figure S9. Amorphous aggregates. Representative TEM images of 100 nM p53WT and p53R248Q formed in chambered slides and in Eppendorf tubes show similar amorphous morphology and size differences. Scale bar = 500 nm

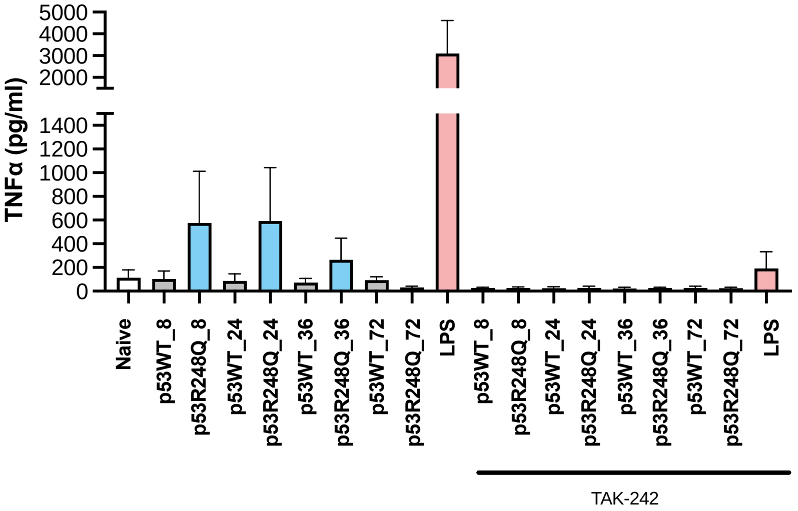

Figure S10. Inflammatory response of aggregates from different time points. Inflammatory response was determined by quantifying TNFα released by macrophages that had been incubated with aggregates (8, 24, 36, 72hr) samples for 24 hours. The conditions were repeated on cells from the same differentiation that had been pre-incubated with the 1 µM TAK-242 (TLR signalling inhibitor) for 1 hour before co-incubating with the test preparations for 24 hours. p53 aggregates were prepared in three separate batches and each batch was assayed on three different Thp1 differentiations which each had 3 technical replicates. Error bars represent standard error of mean.

Supplementary Tables

Table S1: Summary of post-translational modifications of asparagine (Asn), glutamine (Gln), lysine (Lys), methionine (Met), serine (Ser), threonine (Thr) and tyrosine (Tyr) residues found in full length wild-type and mutant p53 derived from bacterial and insect cells from mass spectrometry analysis. Residues within peptides that were not detected during mass spectrometry analysis are denoted as ND (Not Detected).

Residue	Modification	p53WT_ Bacteria	p53WT_ Insect	p53R248Q_ Bacteria	p53R248Q_ Insect	Status in Human cells	Reference
Met 1	Oxidation	✓	✓	✓	✓	Unknown	
Gln 5	Deamidation	✗	✓	✓	✓	Unknown	
Ser 6	Phosphorylation	✗	✓	✗	✓	Known	[1]
Ser 9	Phosphorylation	✗	✓	✗	✓	Known	[1]
Ser 15	Phosphorylation	✗	✓	✗	✓	Known	[2]
Gln 16	Deamidation	✗	✓	✗	✓	Unknown	
Thr 18	Phosphorylation	✗	✗	✗	✗	Known	[3]
Ser 20	Phosphorylation	✗	✗	✗	✗	Known	[1]
Lys 24	Acetylation	✗	✗	✗	✗	Known	[4]
	Methylation	✗	✗	✗	✗	Known	[4]
	Dimethylation	✗	✗	✗	✗	Known	[4]
Asn 29	Deamidation	✓	✓	✓	✓	Known	[5]
Asn 30	Deamidation	✓	✓	✓	✓	Known	[5]
Ser 33	Phosphorylation	✓	✓	✓	✓	Known	[1]
Ser 37	Phosphorylation	✓	✓	✓	✓	Known	[2]
Gln 38	Deamidation	✓	✓	✓	✓	Unknown	
Met 40	Oxidation	✓	✓	✓	✓	Known	[6]
Met 44	Oxidation	✓	✓	✓	✓	Unknown	
Ser 46	Phosphorylation	✓	✓	✓	✓	Known	[7]
Gln 52	Deamidation	✓	✓	✓	✓	Unknown	
Thr 55	Phosphorylation	✓	✓	✓	✗	Known	[8]
Met 66	Oxidation	✓	✓	✓	✓	Unknown	
Thr 81	Phosphorylation	✓	✓	✓	✓	Known	[9]
Ser 90	Phosphorylation	✓	✓	✓	✓	Known	[4]
Ser 94	Phosphorylation	✓	✗	✓	✓	Known	[4]
Ser 95	Phosphorylation	✓	✗	✓	✓	Known	[4]
Ser 96	Phosphorylation	✗	✗	✓	✗	Known	[4]
Ser 99	Phosphorylation	✓	✗	✓	✗	Known	[10]
Gln 100	Deamidation	✓	✓	✓	✓	Unknown	
Lys 101	Acetylation	✓	✓	✓	✓	Known	[4]
	Methylation	✗	✗	✗	✗	Known	[4]
	Dimethylation	✗	✗	✓	✗	Known	[4]

	Trimethylation	✗	✗	✗	✗	Known	[4]
Thr 102	Phosphorylation	✓	✓	✗	✗	Known	[4]
Tyr 103	Phosphorylation	✓	✓	✗	✗	Unknown	
Gln 104	Deamidation	✓	✓	✓	✓	Unknown	
Ser 106	Phosphorylation	✗	✗	✗	✗	Known	[11]
Ser 116	Phosphorylation	✗	✗	✗	✗	Known	[4]
Thr 118	Phosphorylation	✗	✗	✗	✗	Known	[4]
Lys 120	Acetylation	✓	✓	✓	✓	Known	[12]
	Methylation	✗	✗	✗	✗	Known	[4]
	Dimethylation	✗	✗	✗	✗	Known	[4]
	Trimethylation	✗	✗	✗	✗	Known	[4]
Ser 121	Phosphorylation	✗	✗	✗	✓	Known	[4]
Thr 123	Phosphorylation	✗	✗	✗	✗	Known	[4]
Thr 125	Phosphorylation	✗	✗	✗	✗	Known	[4]
Ser 127	Phosphorylation	✗	✗	✗	✗	Known	[4]
Asn 131	Deamidation	✓	✓	✓	✓	Unknown	
Lys 132	Acetylation	✓	✓	✓	✓	Known	[4]
	Methylation	✗	✗	✗	✗	Known	[4]
	Dimethylation	✗	✗	✗	✗	Known	[4]
	Trimethylation	✗	✗	✗	✗	Known	[4]
Met 133	Oxidation	✓	✓	✓	✓	Unknown	
Gln 136	Deamidation	✓	✓	✓	✓	Unknown	
Lys 139	Acetylation	✓	✓	✓	✓	Known	[4]
	Methylation	✗	✗	✗	✗	Known	[4]
	Dimethylation	✗	✗	✗	✗	Known	[4]
	Trimethylation	✗	✗	✗	✗	Known	[4]
Thr 140	Phosphorylation	✓	✗	✓	✓	Known	[4]
Gln 144	Deamidation	✓	✓	✓	✓	Unknown	
Ser 149	Phosphorylation	✗	✓	✓	✓	Known	[13]
Thr 150	Phosphorylation	✗	✓	✗	✓	Known	[13]
Thr 155	Phosphorylation	✗	✗	✗	✗	Known	[13]
Met 160	Oxidation	✓	✓	✓	✓	Known	[6]
Met 169	Oxidation	✓	✓	✓	✓	Unknown	
Tyr 163	Phosphorylation	✗	✗	✓	✓	Unknown	
Lys 164	Acetylation	✓	✓	✓	✓	Known	[14]
	Methylation	✗	✗	✗	✗	Known	[4]
	Dimethylation	✗	✗	✓	✗	Known	[4]
	Trimethylation	✗	✗	✗	✗	Known	[4]
Gln 165	Deamidation	✓	✓	✓	✓	Unknown	
Ser 166	Phosphorylation	✗	✗	✓	✓	Known	[4]
Gln 167	Deamidation	✓	✓	✓	✓	Unknown	
Thr 170	Phosphorylation	✗	✗	✗	✗	Known	[4]

Ser 183	Phosphorylation	✗	✓	✗	✗	Known	[15]
Ser185	Phosphorylation	✗	✓	✗	✗	Known	[4]
Gln 192	Deamidation	✓	✓	✓	✗	Unknown	
Asn 200	Deamidation	✓	✓	✓	✓	Unknown	
Asn 210	Deamidation	✓	✓	✓	✓	Unknown	
Thr 211	Phosphorylation	✗	✗	✗	✗	Known	[16]
Ser 215	Phosphorylation	✗	✗	✗	✗	Known	[17]
Ser 227	Phosphorylation	✓	✓	ND	ND	Known	[4]
Thr 230	Phosphorylation	✓	✓	ND	ND	Known	[4]
Thr 231	Phosphorylation	✓	✓	ND	ND	Known	[4]
Tyr 234	Phosphorylation	✓	✓	ND	ND	Unknown	
Asn 235	Deamidation	✓	✓	ND	ND	Unknown	
Tyr 236	Phosphorylation	✓	✓	ND	ND	Unknown	
Met 237	Oxidation	✓	✓	ND	ND	Unknown	
Asn 239	Deamidation	✓	✓	ND	ND	Unknown	
Ser 240	Phosphorylation	✓	✓	ND	ND	Known	[4]
Ser 241	Phosphorylation	✓	✓	ND	ND	Known	[4]
Met 243	Oxidation	✓	✓	ND	ND	Unknown	
Met 246	Oxidation	✓	✓	ND	ND	Unknown	
Asn 247	Deamidation	✓	✓	ND	ND	Unknown	
Thr 253	Phosphorylation	✗	✗	✗	✗	Known	[4]
Thr 256	Phosphorylation	✗	✗	✗	✗	Known	[4]
Ser 260	Phosphorylation	✗	✗	✗	✗	Known	[4]
Ser 261	Phosphorylation	✗	✗	✗	✗	Known	[4]
Ser 269	Phosphorylation	✗	✗	✗	✗	Known	[4]
Thr 284	Phosphorylation	✗	✗	✗	✗	Known	[15]
Lys 291	Acetylation	✗	✗	✗	✗	Known	[4]
	Methylation	✗	✓	✗	✗	Known	[4]
	Dimethylation	✗	✗	✗	✗	Known	[4]
	Trimethylation	✗	✗	✗	✗	Known	[4]
Lys 292	Acetylation	✗	✓	✓	✓	Known	[18]
	Methylation	✗	✓	✓	✓	Known	[4]
	Dimethylation	✗	✗	✗	✗	Known	[4]
	Trimethylation	✗	✗	✗	✗	Known	[4]
Ser 303	Phosphorylation	✗	✗	✗	✗	Known	[4]
Thr 304	Phosphorylation	✗	✗	✗	✗	Known	[4]
Lys 305	Acetylation	✗	✗	✗	✗	Known	[18]
	Methylation	✗	✗	✗	✗	Known	[4]
	Dimethylation	✗	✗	✓	✗	Known	[4]
	Trimethylation	✗	✗	✗	✗	Known	[4]
Asn 310	Deamidation	✓	✓	✓	✓	Unknown	
Asn 311	Deamidation	✓	✓	✓	✓	Unknown	

Thr 312	Phosphorylation	✗	✗	✗	✗	Known	[19]
Ser 313	Phosphorylation	✗	✗	✗	✓	Known	[19]
Ser 314	Phosphorylation	✗	✓	✗	✓	Known	[19]
Ser 315	Phosphorylation	✗	✓	✗	✓	Known	[2]
Gln 317	Deamidation	✓	✓	✓	✓	Unknown	
Lys 319	Acetylation	✓	✗	✓	✓	Known	[20]
	Methylation	✗	✗	✗	✗	Known	[4]
	Dimethylation	✗	✗	✗	✗	Known	[4]
	Trimethylation	✗	✗	✗	✗	Known	[4]
Lys 320	Acetylation	✓	✗	✗	✗	Known	[21]
	Methylation	✗	✗	✓	✓	Known	[4]
	Dimethylation	✗	✗	✗	✗	Known	[4]
	Trimethylation	✗	✗	✗	✗	Known	[4]
Lys 321	Acetylation	✓	✓	✓	✓	Known	[4]
	Methylation	✗	✗	✓	✓	Known	[4]
	Dimethylation	✗	✗	✓	✓	Known	[4]
	Trimethylation	✗	✗	✗	✗	Known	[4]
Tyr 327	Phosphorylation	✓	✓	✓	✓	Unknown	
Thr 329	Phosphorylation	✗	✗	✗	✓	Known	[4]
Met 340	Oxidation	✓	✓	✓	✓	Unknown	
Asn 345	Deamidation	✓	✓	✓	✓	Unknown	
Lys 351	Acetylation	✓	✓	✗	✓	Known	[4]
	Methylation	✗	✗	✗	✓	Known	[4]
	Dimethylation	✗	✗	✓	✓	Known	[4]
	Trimethylation	✗	✗	✓	✗	Known	[4]
Gln 354	Deamidation	✓	✓	✓	✓	Unknown	
Lys 357	Acetylation	✗	✗	✓	✓	Known	[22]
	Methylation	✗	✗	✗	✓	Known	[4]
	Dimethylation	✗	✗	✗	✓	Known	[4]
	Trimethylation	✗	✗	✗	✗	Known	[4]
Ser 362	Phosphorylation	✗	✗	✗	✗	Known	[23]
Ser 366	Phosphorylation	✗	✗	✗	✗	Known	[24]
Ser 367	Phosphorylation	✗	✗	✗	✗	Known	[4]
Lys 370	Acetylation	ND	ND	ND	ND	Known	[22]
	Methylation	ND	ND	ND	ND	Known	[25]
	Dimethylation	ND	ND	ND	ND	Known	[25]
	Trimethylation	ND	ND	ND	ND	Known	[25]
Ser 371	Phosphorylation	✗	✗	✗	✗	Known	[26]
Lys 372	Acetylation	ND	ND	ND	ND	Known	[27]
	Methylation	ND	ND	ND	ND	Known	[28]
	Dimethylation	ND	ND	ND	ND	Known	[29]
	Trimethylation	ND	ND	ND	ND	Known	[4]

Lys 373	Acetylation	ND	ND	ND	ND	Known	[27]
	Methylation	ND	ND	ND	ND	Known	[30]
	Dimethylation	ND	ND	ND	ND	Known	[30]
	Trimethylation	ND	ND	ND	ND	Known	[4]
Ser 376	Phosphorylation	✗	✗	✗	✗	Known	[26]
Thr 377	Phosphorylation	✗	✗	✗	✗	Known	[26]
Ser 378	Phosphorylation	✗	✗	✗	✗	Known	[26]
Lys 381	Acetylation	ND	ND	ND	ND	Known	[27]
	Methylation	ND	ND	ND	ND	Known	[4]
	Dimethylation	ND	ND	ND	ND	Known	[4]
	Trimethylation	ND	ND	ND	ND	Known	[4]
Lys 382	Acetylation	✗	ND	ND	ND	Known	[22]
	Methylation	✗	ND	ND	ND	Known	[31]
	Dimethylation	✗	ND	ND	ND	Known	[32]
	Trimethylation	✗	ND	ND	ND	Known	[4]
Met 384	Oxidation	✓	ND	✓	ND	Unknown	
Lys 386	Acetylation	✗	ND	✗	ND	Known	[33]
	Methylation	✗	ND	✗	ND	Known	[32]
	Dimethylation	✗	ND	✗	ND	Known	[32]
	Trimethylation	✗	ND	✗	ND	Known	[4]
Thr 387	Phosphorylation	✗	✗	✗	✗	Known	[24]
Ser 392	Phosphorylation	✗	✗	✗	✗	Known	[34]

Table S2. BeStSel secondary structure estimation of p53WT and p53R248Q protein from circular dichroism spectra.

**Secondary Structure**		**p53WT (%)**	**p53R248Q (%)**
**Helix**	Helix (regular)	4.1	4.5
	Helix (distorted)	3.7	3.9
**Antiparallel**	Anti (left-twisted)	1.6	5.3
	Anti (relaxed)	16.6	15.2
	Anti (right-twisted)	13.5	12.9
**Parallel**		0	0
**Turn**		13.7	14.1
**Others**		46.8	44.1

Supplementary References

1. Saito, S., et al., *Phosphorylation site interdependence of human p53 post-translational modifications in response to stress.* J Biol Chem, 2003. **278**(39): p. 37536-44.
2. Lees-Miller, S.P., et al., *Human DNA-activated protein kinase phosphorylates serines 15 and 37 in the amino-terminal transactivation domain of human p53.* Mol Cell Biol, 1992. **12**(11): p. 5041-9.
3. Sakaguchi, K., et al., *Damage-mediated phosphorylation of human p53 threonine 18 through a cascade mediated by a casein 1-like kinase. Effect on Mdm2 binding.* J Biol Chem, 2000. **275**(13): p. 9278-83.
4. DeHart, C.J., et al., *Extensive post-translational modification of active and inactivated forms of endogenous p53.* Mol Cell Proteomics, 2014. **13**(1): p. 1-17.
5. Lee, J.C., et al., *Protein L-isoaspartyl methyltransferase regulates p53 activity.* Nat Commun, 2012. **3**: p. 927.
6. Lu, J., et al., *MICAL2 Mediates p53 Ubiquitin Degradation through Oxidating p53 Methionine 40 and 160 and Promotes Colorectal Cancer Malignance.* Theranostics, 2018. **8**(19): p. 5289-5306.
7. Bulavin, D.V., et al., *Phosphorylation of human p53 by p38 kinase coordinates N-terminal phosphorylation and apoptosis in response to UV radiation.* EMBO J, 1999. **18**(23): p. 6845-54.
8. Gatti, A., et al., *Phosphorylation of human p53 on Thr-55.* Biochemistry, 2000. **39**(32): p. 9837-42.
9. Buschmann, T., et al., *Jun NH2-terminal kinase phosphorylation of p53 on Thr-81 is important for p53 stabilization and transcriptional activities in response to stress.* Mol Cell Biol, 2001. **21**(8): p. 2743-54.
10. Matsuoka, S., et al., *ATM and ATR substrate analysis reveals extensive protein networks responsive to DNA damage.* Science, 2007. **316**(5828): p. 1160-6.
11. Hsueh, K.W., et al., *A novel Aurora-A-mediated phosphorylation of p53 inhibits its interaction with MDM2.* Biochim Biophys Acta, 2013. **1834**(2): p. 508-15.
12. Sykes, S.M., et al., *Acetylation of the p53 DNA-binding domain regulates apoptosis induction.* Mol Cell, 2006. **24**(6): p. 841-51.
13. Bech-Otschir, D., et al., *COP9 signalosome-specific phosphorylation targets p53 to degradation by the ubiquitin system.* EMBO J, 2001. **20**(7): p. 1630-9.
14. Tang, Y., et al., *Acetylation is indispensable for p53 activation.* Cell, 2008. **133**(4): p. 612-26.
15. Wu, L., et al., *Aurora B interacts with NIR-p53, leading to p53 phosphorylation in its DNA-binding domain and subsequent functional suppression.* J Biol Chem, 2011. **286**(3): p. 2236-44.
16. Gully, C.P., et al., *Aurora B kinase phosphorylates and instigates degradation of p53.* Proc Natl Acad Sci U S A, 2012. **109**(24): p. E1513-22.

17. Liu, Q., et al., *Aurora-A abrogation of p53 DNA binding and transactivation activity by phosphorylation of serine 215.* J Biol Chem, 2004. **279**(50): p. 52175-82.

18. Wang, Y.H., et al., *Identification and characterization of a novel p300-mediated p53 acetylation site, lysine 305.* J Biol Chem, 2003. **278**(28): p. 25568-76.

19. Yang, F., et al., *Phosphoproteome profiling of human skin fibroblast cells in response to low- and high-dose irradiation.* J Proteome Res, 2006. **5**(5): p. 1252-60.

20. Abraham, J., et al., *Post-translational modification of p53 protein in response to ionizing radiation analyzed by mass spectrometry.* J Mol Biol, 2000. **295**(4): p. 853-64.

21. Sakaguchi, K., et al., *DNA damage activates p53 through a phosphorylation-acetylation cascade.* Genes Dev, 1998. **12**(18): p. 2831-41.

22. Joubel, A., et al., *Identification of new p53 acetylation sites in COS-1 cells.* Mol Cell Proteomics, 2009. **8**(6): p. 1167-73.

23. Xia, Y., et al., *Phosphorylation of p53 by IkappaB kinase 2 promotes its degradation by beta-TrCP.* Proc Natl Acad Sci U S A, 2009. **106**(8): p. 2629-34.

24. Ou, Y.H., et al., *p53 C-terminal phosphorylation by CHK1 and CHK2 participates in the regulation of DNA-damage-induced C-terminal acetylation.* Mol Biol Cell, 2005. **16**(4): p. 1684-95.

25. Huang, J., et al., *p53 is regulated by the lysine demethylase LSD1.* Nature, 2007. **449**(7158): p. 105-8.

26. Lu, H., et al., *The CDK7-cycH-p36 complex of transcription factor IIH phosphorylates p53, enhancing its sequence-specific DNA binding activity in vitro.* Mol Cell Biol, 1997. **17**(10): p. 5923-34.

27. Gu, W. and R.G. Roeder, *Activation of p53 sequence-specific DNA binding by acetylation of the p53 C-terminal domain.* Cell, 1997. **90**(4): p. 595-606.

28. Chuikov, S., et al., *Regulation of p53 activity through lysine methylation.* Nature, 2004. **432**(7015): p. 353-60.

29. Huang, J., et al., *Repression of p53 activity by Smyd2-mediated methylation.* Nature, 2006. **444**(7119): p. 629-32.

30. Huang, J., et al., *G9a and Glp methylate lysine 373 in the tumor suppressor p53.* J Biol Chem, 2010. **285**(13): p. 9636-9641.

31. Shi, X., et al., *Modulation of p53 function by SET8-mediated methylation at lysine 382.* Mol Cell, 2007. **27**(4): p. 636-46.

32. Kachirskaia, I., et al., *Role for 53BP1 Tudor domain recognition of p53 dimethylated at lysine 382 in DNA damage signaling.* J Biol Chem, 2008. **283**(50): p. 34660-6.

33. Li, A.G., et al., *An acetylation switch in p53 mediates holo-TFIID recruitment.* Mol Cell, 2007. **28**(3): p. 408-21.

34. Sakaguchi, K., et al., *Phosphorylation of serine 392 stabilizes the tetramer formation of tumor suppressor protein p53.* Biochemistry, 1997. **36**(33): p. 10117-24.
