# Supplementary material for: Characterization of full-length p53 aggregates and their kinetics of formation
Source: Biophys J. 2022 Oct 13;121(22):4280–98. doi: 10.1016/j.bpj.2022.10.013 (PMC9703098; doi:10.1016/j.bpj.2022.10.013)
Supplement: Document S2. Article plus supporting material [file mmc2.pdf]

Biophysical Journal
Article

Characterization of full-length p53 aggregates and their kinetics of formation

Linda Julian,[1,2] Jason C. Sang,[2] Yunzhao Wu,[2] Georg Meisl,[2] Jack H. Brelstaff,[3,4] Alyssa Miller,[5] Matthew R. Cheetham,[2] Michele Vendruscolo,[5] Tuomas P. J. Knowles,[5] Francesco Simone Ruggeri,[5] Clare Bryant,[4,7] Susana Ros,[1] Kevin M. Brindle,[1,*] and David Klenerman[2,8,*]

[1]Cancer Research UK Cambridge Institute, University of Cambridge, Cambridge, United Kingdom; [2]Yusuf Hamied Department of Chemistry, University of Cambridge, Cambridge, United Kingdom; [3]Department of Clinical Neurosciences, University of Cambridge, Cambridge, United Kingdom; [4]Department of Medicine, University of Cambridge, Cambridge, United Kingdom; [5]Centre for Misfolding Diseases, Yusuf Hamied Department of Chemistry, University of Cambridge, Cambridge, United Kingdom; [7]Department of Veterinary Medicine, University of Cambridge, United Kingdom; and [8]UK Dementia Research Institute, University of Cambridge, Cambridge, United Kingdom

ABSTRACT Mutations in the *TP53* gene are common in cancer with the R248Q missense mutation conferring an increased propensity to aggregate. Previous p53 aggregation studies showed that, at micromolar concentrations, protein unfolding to produce aggregation-prone species is the rate-determining step. Here we show that, at physiological concentrations, aggregation kinetics of insect cell-derived full-length wild-type p53 and p53R248Q are determined by a nucleation-growth model, rather than formation of aggregation-prone monomeric species. Self-seeding, but not cross-seeding, increases aggregation rate, confirming the aggregation process as rate determining. p53R248Q displays enhanced aggregation propensity due to decreased solubility and increased aggregation rate, forming greater numbers of larger amorphous aggregates that disrupt lipid bilayers and invokes an inflammatory response. These results suggest that p53 aggregation can occur under physiological conditions, a rate enhanced by R248Q mutation, and that aggregates formed can cause membrane damage and inflammation that may influence tumorigenesis.

SIGNIFICANCE More than half of all human cancers carry mutations in p53, and the R248Q hotspot mutation increases the aggregation propensity of the protein. To investigate aggregation kinetics, we monitored aggregation of wild-type and mutant p53R248Q at sub-micromolar concentrations using full-length proteins prepared from baculovirus-infected insect cells. We show that, at physiologically relevant concentrations, the increased aggregation propensity of R248Q mutation was due to decreased solubility and increased aggregation rate, leading to the formation of a greater number of larger amorphous aggregates. Our data suggest that, if enough aggregates are formed in vivo, they would be capable of altering the local environment around a cell with p53R248Q mutation, which could have important implications for cancer pathogenesis.

INTRODUCTION

Aggregated proteins are of central importance in the pathology of many neurodegenerative diseases including Alzheimer's disease and Parkinson's disease, as well as in other disorders including cystic fibrosis and type II diabetes (1). Most proteins contain one or more short aggregation-prone regions (APRs) that tend to be buried in the hydrophobic core of the protein where they are prevented from initiating aggregation (2) in normally folded proteins. Nevertheless, mutations that destabilize the native conformation can increase the probability of these APRs becoming solvent exposed and self-assembling by intermolecular β sheet interactions to form the β sheet-rich core of a nascent aggregate. An in silico survey showed that 38% of mutations found in cancer can also result in an increased aggregation propensity of the affected protein (2). The tumor suppressor p53 was one of the candidates identified with possible

Submitted April 12, 2022, and accepted for publication October 11, 2022.

*Correspondence: kmb1001@cam.ac.uk or dk10012@cam.ac.uk

Francesco Simone Ruggeri's present address is Laboratory of Organic Chemistry, Wageningen University & Research, Wageningen, the Netherlands.

Francesco Simone Ruggeri's present address is Laboratory of Physical Chemistry, Wageningen University & Research, Wageningen, the Netherlands.

Editor: Sudipta Maiti.

https://doi.org/10.1016/j.bpj.2022.10.013

© 2022 Biophysical Society.

This is an open access article under the CC BY license (http://creativecommons.org/licenses/by/4.0/).

aggregation-inducing mutations (2). Indeed, abnormal accumulation of p53 aggregates have been described in several types of cancer, including bladder carcinoma, neuroblastoma, and breast and colorectal cancers (3–6). More than half of all human cancers contain one or more mutations in p53 (7), underlining its importance as a tumor suppressor by both blocking cell cycle progression and promoting apoptotic cell death (8). The 393-amino acid protein has limited stability and contains largely unstructured regions in the N- and C-terminal domains, whereas the DNA-binding domain and tetramerization domain adopt well-defined secondary structures (9,10).

More than 90% of cancer-causing mutations occur in the core DNA-binding domain of p53, and some of these can alter the protein structure, thus leading to misfolding and aggregation. The mutation R248 is located in a DNA-binding position that is frequently altered in cancer. Earlier studies have shown that the R248Q mutant exhibits an enhanced propensity to aggregate compared with p53 wild-type (p53WT) (4). However, the majority of aggregation studies on wild-type and mutant p53 in vitro have focused on specific domains of the protein, including the core DNA-binding domain, transactivation domain, and tetramerization domain (11–18), with the full-length protein receiving only limited attention (4,19,20). Since the aggregation kinetics of full-length p53 were found to be very similar to that of its core domain (4,19), this has been used to support the use of wild-type and mutant p53 core domains for most aggregation studies. However, while many tumor-suppressor genes are inactivated by deletions, truncating mutations, or epigenetic mechanisms, missense mutations leading to expression of functionally altered full-length mutant proteins with single amino acid substitutions comprise approximately 75% of all p53 alterations, with less than 10% of cancers harboring a truncated form of the protein (21), thus emphasizing the importance of studying the full-length protein.

Investigations on the mechanisms of p53 aggregation found that the kinetics at micromolar concentrations are usually dominated by protein unfolding to produce an aggregation-competent precursor, rather than the aggregation reaction itself (19,22). Thus, little information about the mechanism of p53 aggregation could be gained from these experiments. Moreover, previous aggregation studies, including those that showed that aggregation of the full-length p53 protein was similar to that of the core domain (4,19), have relied on recombinant protein expressed in *Escherichia coli*, which may lack some key post-translational modifications that would be present in the human proteins (23) and that could affect its aggregation properties (24). To avoid the potential shortcomings of using bacterial systems for recombinant protein expression, and to investigate the mechanism of aggregation itself, we monitored the aggregation of p53 at sub-micromolar concentrations using proteins prepared from baculovirus-infected insect cells that carried the majority of the post-translational modifications found in mammalian cells. This allowed us to monitor aggregation in a regime where the kinetics is dominated by the aggregation reaction rather than the formation of an aggregation-prone species. We then explored the biophysical characteristics of both full-length p53WT and p53R248Q aggregates using a range of methods. Our results show that p53 can aggregate at physiological concentrations, forming aggregates that can damage membranes and invoke an inflammatory response.

METHODS

Protein expression and purification

Bacteria

E. coli OverExpress C41 (DE3) competent cells (Lucigen) were transformed with pRSET-HLT plasmids (kind gift from Prof. A.R. Fersht, MRC-LMB Cambridge) containing full-length p53WT. The mutant p53R248Q version of the plasmid was generated by site-directed mutagenesis using the following primers: R248Q-F, 5′GGCGGCATGAACC AGAGGCCCATCCTC and R248Q-R, 5′GAGGATGGGCCTCTGGTT CATGCCGCC. Cells were grown at 37°C in 2xYT medium to an optical density (600 nm) of 0.6 and induced with 1 mM isopropyl-thio-galactopyranoside (IPTG). Zinc sulfate, at a final concentration of 0.1 mM, was also added to the culture. After incubation for 16 h at 22°C, the cells were harvested by centrifugation (Avanti J-26 XPI, Beckman Coulter) at 4500 rpm for 20 min at 4°C.

Cell pellets were resuspended in lysis buffer containing 50 mM potassium phosphate, pH 8.0, 300 mM NaCl, 10 mM imidazole, 15 mM β-mercaptoethanol, together with one protease inhibitor tablet (cOmplete, EDTA-free, Merck) per 10 mL of lysis buffer. Cells were lysed by passing the lysate twice through a high-pressure homogenizer (Avestin Emulsiflex C5, ATA Scientific Instruments) at approximately 15,000 psi. The cell lysate was cleared of debris by centrifugation at 16,000 rpm for 30 min at 4°C and the supernatant filtered through a 0.45-μm filter (Millipore). The crude lysate was passed through a HisTrap FF Crude column (GE Healthcare) and eluted with a step gradient of the elution buffer (50 mM potassium phosphate, pH 8.0, 300 mM NaCl, 250 mM imidazole, 15 mM β-mercaptoethanol) using a fast protein liquid chromatography (FPLC) system (AKTA Explorer, GE Healthcare). Eluted fractions were pooled and digested overnight at 4°C with Tobacco Etch Virus (TEV) protease (1 mg of TEV/100 mg protein) to cleave the HLT tag. The digested fractions were diluted in heparin binding buffer containing 25 mM phosphate, pH 7.5, 5 mM dithiothreitol (DTT, Merck), and 10% glycerol and the fractions passed through a HiTrap Heparin HP column (GE Healthcare) eluted with a step gradient of the elution buffer (25 mM potassium phosphate, pH 7.5, 1 M NaCl, 5 mM DTT, and 10% glycerol). The eluted fractions were further purified using a HiLoad 26/60 Superdex 200 prep grade size exclusion column (GE Healthcare) and eluted in 25 mM phosphate, pH 7.2, 300 mM NaCl, 5 mM DTT, and 10% glycerol. All buffers used for purification were made using Milli-Q water and filtered through a 0.22-μm filter (Stericup Quick Release, Millipore). β-Mercaptoethanol and DTT were added immediately before use. Proteins were aliquoted in small volumes to minimize freeze-thaw cycles, flash frozen, and stored at −80°C in 50 mM Tris-HCl, 500 mM NaCl, 5% glycerol, pH 8.0. Aliquots were thawed once on ice before use. Proteins were analyzed by SDS-PAGE and western blotting with anti-p53 antibody (DO-1, Santa Cruz Biotechnology) for molecular weight and purity measurements.

Insects

All p53 protein samples were expressed and purified from insect cells by GenScript Biotech (Netherlands).

Gene synthesis and subcloning

Target DNA sequences encoding p53 wild type (p53WT) and p53R248Q were synthesized and then subcloned into pFastBac1 for insect cell expression. DH10Bac strain was used for recombinant bacmid (rbacmid) generation. The positive rbacmid was confirmed by PCR. *Spodoptera frugiperda* (Sf9) cells were grown in Sf-900 II SFM Expression Medium (Life Technologies) in Erlenmeyer flasks at 27°C in an orbital shaker. One day before transfection, the cells were seeded at an appropriate density in six-well plates. Plasmid DNA and Transfection Reagent (PROMEGA) were mixed and then added to the plate with cells ready for transfection. Cells were incubated in Sf-900 II SFM for 5–7 days at 27°C before harvest. The supernatant was collected after centrifugation and designated as progeny 1 (P1) viral stock. P2 was amplified for later infection.

Expression and purification of p53 proteins

Sf9 cells were infected with P2 virus and incubated in Sf-900II SFM(1X) for 2–3 days at 27°C. Cell pellets were collected by centrifugation and target protein was purified by affinity chromatography and ion exchange chromatography. Higher-purity fractions were pooled and followed by 0.22-μm filter sterilization. The concentration was determined by Bradford protein assay with bovine serum albumin (BSA) as a standard. Proteins were aliquoted in small volumes to minimize freeze-thaw cycles, flash frozen, and stored at −80°C in 50 mM Tris-HCl, 500 mM NaCl, 5% glycerol, pH 8.0. Aliquots were thawed once on ice before use.

Preparation of aggregation buffer and thioflavin T

Aggregation assays were performed in 50 mM Tris, pH 7.2, containing 5 mM DTT and 150 mM NaCl (aggregation buffer). DTT was added just before use, followed by filtration through a 0.02-μm syringe filter (Anotop 25 inorganic membrane filter, Whatman). The aggregation buffer was freshly prepared before each experiment and maintained at 4°C until use.

Thioflavin T (ThT; Sigma Aldrich) stock solution was prepared by dissolving a small amount in absolute ethanol by vigorous vortexing and then diluting in phosphate-buffered saline (PBS) to a concentration of around 500 μM. The solution was then filtered using a 0.02-μm syringe filter. The exact concentration of ThT was determined by UV absorbance (Nanodrop 2000, Thermo; ε421 nm = 36,000 $M^{-1}cm^{-1}$) and 1 μM ThT working solution was prepared by diluting in filtered aggregation buffer.

Single-aggregate imaging with thioflavin T

Monomer aggregation

Monomeric p53WT and p53R248Q proteins that were stored −80°C were defrosted and rapidly diluted in aggregation buffer containing 1 μM ThT. Thirty microliters of monomer solution was transferred to the center of each well of an eight-well glass-bottom chambered slide (Ibidi, μ-Slide, catalog no. 80827), forming a droplet that did not contact the walls of the well. The slide was then sealed with Parafilm and loaded onto a home-built microscope (see below) with a thermally controlled chamber (37°C and ~50% humidity) for 72 h.

Seeded aggregation

Seeds were prepared by aggregating 100 nM monomeric proteins in an eight-well glass-bottom chamber and incubating at 37°C and ~50% humidity for 8 h. Residual monomers in the droplet were removed by washing with the aggregation buffer five times so that only the preformed seeds remained on the surface of the glass-bottom chamber. This setup allowed for seed generation under physiological conditions and without any external perturbation such as sonication. The presence of seeds was confirmed by imaging before adding 100 nM monomer samples containing 1 μM ThT. Homologous (self-seeded) aggregations were set up with p53WT/p53R248Q seeds with 100 nM p53WT/p53R248Q monomer respectively. Heterologous (cross-seeded) aggregations were set up using p53WT seeds with p53R248Q monomers and p53R248Q seeds with p53WT monomers. The slide was then sealed with Parafilm and loaded onto a home-built microscope with a thermally controlled chamber (37°C and ~50% humidity) for 72 h.

Imaging p53 aggregation

Imaging was performed on a home-built microscope with epi-illumination using a 40× objective (Nikon Plan Fluor, NA = 0.75) installed on an inverted microscope (Nikon, Eclipse Ti-E). A 405-nm laser (Oxxius, LBX-LD) at 35 mW was used as the light source to illuminate the samples with epi-illumination. The microscope was fitted with a perfect focus system that auto-corrects the z-stage drift during a prolonged period of imaging. The laser power was attenuated by neutral density (ND) filters (ND = 0) before the beam was passed through a quarter-wave plate (to circularly polarize the laser beam), a beam expander, and their respective excitation filters (FF01-417/60-25 for 405 nm). The lasers were combined by a dichroic mirror (Di02-R442–25x36, Semrock), passed through the back port of the microscope, and focused on the sample by the objective. Fluorescence was collected by the objective and separated from the excitation light by a dichroic mirror (ZT R405/532 RPC, Semrock), and passed through appropriate filters (FF01-480/40–25 for ThT). The 512 × 512-pixel images were obtained with an electron-multiplying charge-coupled device (EMCCD) camera (Evolve 512, Photometrics) with a pixel size of 322 nm. Images were acquired continually at fixed fields of view (FOVs) using an automated script (Micro-Manager) to prevent user bias. Exposure times were kept constant at 50 ms, and 50 frames were acquired for diffraction-limited images. For each well, images were acquired continually from nine (3 × 3) FOVs, with the horizontal and vertical gaps between adjacent FOVs set at 200 μm.

Image processing

Individual images were averaged over all frames using ImageJ to suppress random noise and then analyzed with custom-written MATLAB scripts (R2020a, Mathworks). Images were bandpass filtered to remove the modulated background and camera noise and then blurred using a 2D-Gaussian filter. Particle boundaries were identified by fine-tuning size and intensity threshold while their positions were located by calculating centroid positions. To eliminate the background effect in intensity calculation, the signal-to-background-ratio (SBR) was used to correct the intensity of pixels. SBR is defined as the intensity above the background over the background. For a given particle, its corrected intensity is the sum of SBR of each pixel within its boundary.

Determination of soluble total monomer concentration

The 20-μL aliquots were collected from the droplet at the start of aggregation and after 72 h. The aliquots were mixed with NuPAGE LDS sample buffer (Invitrogen) and NuPAGE sample reducing agent (Invitrogen) and heated for 5 min at 95°C. Samples were loaded onto the NuPAGE 4%–12% Bis-Tris gel, 1.0 mm (Invitrogen) and electrophoresis was performed at 100 V for the initial 10 min followed by 200 V until the dye front reached the bottom of the gel. Proteins were transferred to a nitrocellulose membrane (iBlot, Invitrogen), blocked for 1 h in Intercept Blocking Buffer PBS (LI-COR), and then incubated for 1 h at room temperature with primary antibody anti-p53 (DO-1, Santa Cruz) diluted 1:1000 with Intercept Blocking Buffer. After three washes with PBS containing 1% Tween (PBS-T), membrane was incubated with secondary antibody IRDye 800-conjugated goat anti-mouse IgG diluted 1:5000 in blocking buffer. Membrane was washed three times with PBS-T and final wash with PBS before scanning using Odyssey CLx (LI-COR). Known p53

standards were also immunoblotted to prepare a standard curve, thus allowing quantitative estimation of p53 monomer before and after 72 h of aggregation.

Proteinase K sensitivity assay

Recombinant Proteinase K (PK, Invitrogen) stock solution was prepared at a concentration of 50 μM in PBS. Monomer solutions were aggregated as a droplet in Ibidi glass-bottomed chambered slides (eight wells) at 37°C in a humidified chamber. At defined time points, residual monomers in the droplet were removed by washing with the aggregation buffer five times so that only the preformed seeds remained on the surface of the glass-bottom chamber. To this, aggregation buffer containing 1 μM ThT was added. The slide was loaded onto a microscopic stage that was maintained at 37°C. Depending on the number of aggregates present for each sample, 49–144 images were taken for each droplet. Once imaging was completed, PK was added to the droplet at a final concentration of 0.5 μM and mixed well. End-of-reaction images at the fixed FOVs were also recorded.

Mass spectrometry

Monomer (5 g) was resuspended in sample buffer containing 1× NuPAGE LDS sample buffer (Invitrogen) and 1× NuPAGE sample reducing agent (Invitrogen) and heated for 5 min at 95°C. Samples were loaded onto the NuPAGE 4%–12% Bis-Tris gel (1.0 mm, 10 well) (Invitrogen) and electrophoresis was performed using the MOPS Running buffer (Invitrogen) at 100 V for the initial 10 min followed by 200 V until the dye front reached the bottom of the gel. The gel was stained with InstantBlue Coomassie protein stain (Abcam) for 1 h at room temperature. After one-dimensional electrophoresis, the gel was rinsed with water for a few hours before excising the bands of interest. The excised bands were destained using 100% acetonitrile and 100 mM ammonium bicarbonate and dehydrated in acetonitrile for 10 min to remove all the liquid and buffer from the gel pieces. Disulfide bonds were reduced by incubating in 150 μL of 10 mM DTT in 100 mM ammonium bicarbonate for 1 h at 56°C. The gel pieces were washed with 150 μL of 10 mM DTT in 100 mM ammonium bicarbonate and the free thiol groups alkylated with 100 mM iodoacetamide in 100 mM ammonium bicarbonate by incubating for 45 min in the dark at room temperature. The gel pieces were then dehydrated in acetonitrile before digestion with Glu-C (Promega, V1651) followed by trypsin (Thermo 90058) or trypsin alone. Gel pieces were incubated with 10 μL of 100-ng Glu-C for 4 h at 37°C followed by 10 μL of 100-ng trypsin in 100 mM ammonium bicarbonate or trypsin alone for 30 min at 4°C. An additional 20 μL of 100 mM ammonium bicarbonate was added to the gel pieces and digested overnight at 37°C. Next day, the first eluate (supernatant) and the second eluate obtained by dehydrating the gel pieces with acetonitrile were pooled. The eluate was acidified with formic acid and then dried completely by vacuum centrifugation.

For liquid chromatography-tandem mass spectrometry (LC-MS/MS) analysis, each dried peptide sample was reconstituted in 10 μL of 0.1% (v/v) formic acid. Peptides were loaded and separated on a reverse-phase trap column (Thermo 164213, 5-μm C18 particles, 2 cm [100-μm internal diameter (i.d.)]) and analytical column (Thermo ES902, 2-μm C18 particles, 25 cm [75-μm i.d.]) respectively using a 5%–45% acetonitrile gradient in 0.1% formic acid and 5% DMSO at 300-nL/min flow rate. In each data collection cycle, one full MS scan (400–1600 m/z) was acquired in an Orbitrap Fusion Lumos (Thermo) with the following settings: 60,000 resolution, automatic gain control (AGC) of 3×10^6, and maximum injection time (MIT) of 200 ms. The most abundant ions were selected for fragmentation by collision-induced dissociation (CID). CID was performed with a collision energy of 28%, an AGC setting of 5 × 104, an isolation window of 2.0 Da, and an MIT of 300 ms. Previously analyzed precursor ions were dynamically excluded for 40 s.

Identification of p53 post-translational modifications

The Proteome Discoverer 2.1. (Thermo Scientific) was used for processing CID tandem mass spectra. The SequestHT search engine was used and all the spectra searched against the Uniprot Homo sapiens FASTA database (taxon ID 9606, version February 2019) with the enzymes set to Glu-C plus trypsin (semi) or trypsin (full). All searches were performed as a static modification of carbamidomethyl at cysteines (+57.051 Da). Methionine oxidation (+15.9949 Da); deamidation on asparagine and glutamine (+0.984); phosphorylation on serine, threonine, and tyrosine (+79.066); ubiquitination (+114.042) on lysine, serine, and threonine; and acetylation (+42.0105) and methylation (+14.016) on lysine were included as dynamic modifications. Mass spectra were searched using a precursor ion tolerance of 20 ppm and fragment ion tolerance of 0.02 Da. For peptide confidence, 1% false discovery rate (FDR) was applied and peptides uniquely matched to a protein were used for further analysis.

Transmission electron microscopy

The ultrastructure of p53 aggregates was analyzed using transmission electron microscopy (TEM) in a Talos F200X G2 (Thermo Scientific) TEM at 200 kV using negative staining. Briefly, 300-mesh gold TEM grids (Agar Scientific) were cleaned by glow discharging (GloQube, Quorum) for 15 min and then placed at the center of each well of an eight-well glass-bottom chambered slide (Ibidi). Thirty microliters of protein sample (100 nM of p53WT or p53R248Q) was added to the well to form a droplet with the grid at the center of the droplet. The chambered slide was sealed with Parafilm (Bemis) and incubated in a humidified chamber at 37°C. At specific time points after initiation of aggregation, the grid was taken out of the well, washed twice with 5 μL of filtered Milli-Q water, stained with 5 μL of 2% uranyl acetate for 30 seconds, and washed again twice with 5 μL of filtered Milli-Q water. The residual liquid after each step was removed using a piece of filter paper (Whatman). The grid was then air dried before imaging.

Atomic force microscopy

Wild-type and mutant p53R248Q (100 nM) were aggregated in Protein LoBind tubes (Eppendorf, Sigma) in filtered aggregation buffer by incubating at 37°C in an orbital shaker (New Brunswick Scientific Innova 43) at 200 rpm for 72 h. Solutions containing aggregates (10 μL) were deposited onto freshly cleaved mica that was positively functionalized using (3-aminopropyl) triethoxysilane (APTES; 0.5% w/v), and incubated for 5 min before rinsing with 1 mL of Milli-Q water. The samples were then dried using a gentle flow of nitrogen gas. Atomic force microscopy (AFM) maps were acquired using NX10 AFM (Park Systems, South Korea) operating in non-contact mode. Imaging was performed in a constant phase change regime (25). The setup was equipped with a silicon nitride cantilever (PPP-NCHR) with a nominal tip radius of <10 nm and a spring constant of 5 N/m. Image processing was performed using SPIP software (Image Metrology, Denmark); images were first-order flattened and the height profiles measured. All measurements were performed at room temperature.

Circular dichroism spectroscopy

Solutions of p53WT and p53R248Q were diluted in 10 mM phosphate buffer, pH 7.2, to a concentration of 2.5 μM and transferred to a 0.1-cm path length quartz cuvette (Hellma Macro Cuvettes). CD spectra were recorded from 195 to 260 nm, with a bandwidth of 1 nm, a step size of 1 nm, and a time per point of 0.5 s, at 20°C using a Chirascan Circular Dichroism Spectrometer (Applied Photophysics Limited, UK) with a Peltier temperature control system. For each sample, an average of three

measurements was taken, baseline corrected, and smoothed using the Savitzky-Golay method. Molar ellipticity was calculated using the following formula: molar ellipticity (θ) = millidegrees/(path length in millimeter × the molar concentration of proteins × the number of residues) (26).

Estimation of protein secondary structure from CD spectra was performed using BeStSel (27).

Supported lipid bilayer assay

Preparation of lipid vesicle suspension

A mixture of 40 μL of 25 mg/mL 1-palmitoyl-2-oleoyl-*sn*-glycero-3-phosphocholine (POPC, Avanti) and 14 μL of 1 mg/mL Oregon Green 488 1,2-dihexadecanoyl-*sn*-glycero-3-phosphoethanolamine (OG-DHPE, Invitrogen) in chloroform was prepared in a clean glass vial, dried with a gentle flow of nitrogen, and kept in a vacuum desiccator for 12 h to remove any residual solvent. The lipid mixture was then suspended in 1 mL of Milli-Q water using a vortex mixer. The cloudy lipid suspension was sonicated with a 2-mm titanium probe (Sonicator microprobe 4423, Qsonica) mounted on a tip sonicator (ultrasonic processor Q125, Qsonica) for 20 min in an ice-water bath. The sonicator was operated at 60% maximum power with 45 s/15 s on/off cycles. The lipid suspension turned clear after sonication, indicating formation of small unilamellar lipid vesicles (SUVs). Finally, the SUV suspension was centrifuged at 14,100 × g for 2 min, after which the supernatant was retained to remove the titanium particles from the sonication probe. The suspension was kept at 4°C before use.

Preparation of slides and immobilization of lipid vesicles

Glass coverslips (VWR) were pre-cleaned by sonication in Milli-Q water followed by propan-2-ol and again in Milli-Q water for 5 min each. The coverslips were then treated for 8 min with a freshly prepared solution of a 3:1 mixture of sulfuric acid and 30% hydrogen peroxide, rinsed thoroughly with Milli-Q water and dried with nitrogen. Two 50-well gaskets (Grace Bio-Labs CultureWell, Merck) were stacked and affixed on the coverslip. Fifteen microliters of the SUV suspension (diluted 1:1 with PBS) was added to the wells and incubated at room temperature for 10 min, rinsed three times with Milli-Q water, and then with PBS.

Imaging of SLB

Wild-type and mutant p53 proteins were aggregated in Protein LoBind tubes (Eppendorf, Sigma) in filtered aggregation buffer at 37°C in an orbital shaker (New Brunswick Scientific Innova 43) at 200 rpm for 72 h. The aggregate samples were sonicated (ultrasonic cleaner USC100T, VWR) for 5 min in an ice water bath and briefly mixed using a vortex mixer before use. Solution containing p53 aggregates were added to the wells and sealed with a clean glass coverslip (VWR) on top of the PDMS gasket. The glass coverslip was then fixed onto a customized sample holder for imaging with an oil-immersion objective (CFI Apochromat total internal reflection fluorescence [TIRF] 100XC Oil, Nikon) on an inverted microscope (Eclipse Ti-E, Nikon). A 488-nm laser (Cobolt 06-MLD-488, HÜBNER) was used as the light source and the laser power was set to 70 mW. The laser power was attenuated with a neutral density filter (NE30B-A, Thorlabs) before being introduced into the objective. The coverslip was illuminated with TIRF illumination mode and imaged after 24 h of incubation at 30°C. For each well, nine FOVs (three rows by three columns) were imaged. The horizontal and vertical distances between adjacent rows or columns were set to 150 μm. For each FOV, 50 frames were recorded with an sCMOS camera (Prime BSI Express, Photometrics) with the exposure time for each frame set to 50 ms. The pixel size of the image was 87.21 nm. Averaged images were analyzed for the number and total area of holes with a self-written Python script.

Inflammation assay

Preparation of macrophages

Thp1 cells were maintained as non-adherent monocytes at less than 800,000 cells/mL in RPMI with glutamine and supplemented with 10% heat-inactivated fetal bovine serum (FBS) and 1% antibiotic and antimycotic (10,000 units/mL penicillin G, 10 mg/mL streptomycin, and 25 μg/mL amphotericin B) under 5% (v/v) CO_2 at 37°C. THP-1 cells were then plated at 50,000 cells per well in the presence of 150 nM phorbol 12-myristate 13-acetate (PMA) in a 96-well plate and incubated for 24 h to differentiate these cells into adherent macrophages. PMA medium was aspirated and replaced with RPMI medium supplemented with 10% heat-inactivated FBS and 1% antibiotic and antimycotic for a further 24 h.

Preparation of p53 aggregates

Endotoxin levels of protein solutions were quantified using Pierce LAL Chromogenic Endotoxin Quantitation Kit (Thermo Scientific) using manufacturer's instructions. p53WT and p53R248Q insect-derived proteins were aggregated in Protein LoBind tubes (Eppendorf, Sigma) in filtered aggregation buffer at 37°C in an orbital shaker (New Brunswick Scientific Innova 43) at 200 rpm for 72 h. The aggregate solutions were ultracentrifuged at 90,000 rpm for 1 h at 4°C. Aggregates formed using this method had similar amorphous morphology and size differences to those formed in chambered slides (Fig. S9). The supernatant was discarded and the aggregates were resuspended in RPMI medium supplemented with 10% heat-inactivated FBS.

Treatment of macrophages with p53 aggregates

Medium was replaced with either fresh control medium, 200 ng/mL of ultra-pure lipopolysaccharide (LPS), or one of the following preparations: 1 μM p53WT or p53R248Q in both non-aggregated or aggregated form. Additionally, these conditions were repeated with cells from the same differentiation that had been incubated with the Toll-like receptor (TLR) signaling inhibitor TAK-242 for 1 h at 1 μM, and then co-incubated with the test preparations for 24 h. Conditioned medium was collected and centrifuged at 100× g for 10 min to pellet cellular material and debris. The concentration of tumor necrosis factor alpha (TNFα) was assayed using TNFα DuoSet ELISA. p53 aggregates were prepared in three separate batches and each batch was assayed on three different Thp1 differentiations which each had three technical replicates. Statistical analysis was performed using one-way ANOVA with Dunnett's correction.

RESULTS

Phosphorylation of insect and bacterial cell-derived recombinant p53

Human p53 is composed of 393 amino acid residues and has a modular structure, consisting of a transactivation domain, a proline-rich domain, a DNA-binding domain, a tetramerization domain, and a basic domain (Fig. 1 *c*). We subjected full-length p53WT and mutant p53R248Q purified from bacterial and insect cells to mass spectrometric analysis and identified a number of co-occurring and mutually exclusive post-translational modifications (PTMs) on specific residues. The majority of the differences between the bacterial and insect cell-derived proteins were in the phosphorylation of serine residues (Fig. 1 *a* and *b*). There were 10 phosphorylated Ser residues in both bacterial cell-derived proteins compared with 15 in their insect cell-derived counterparts. In particular, phosphorylation of serine residues S6, S9,

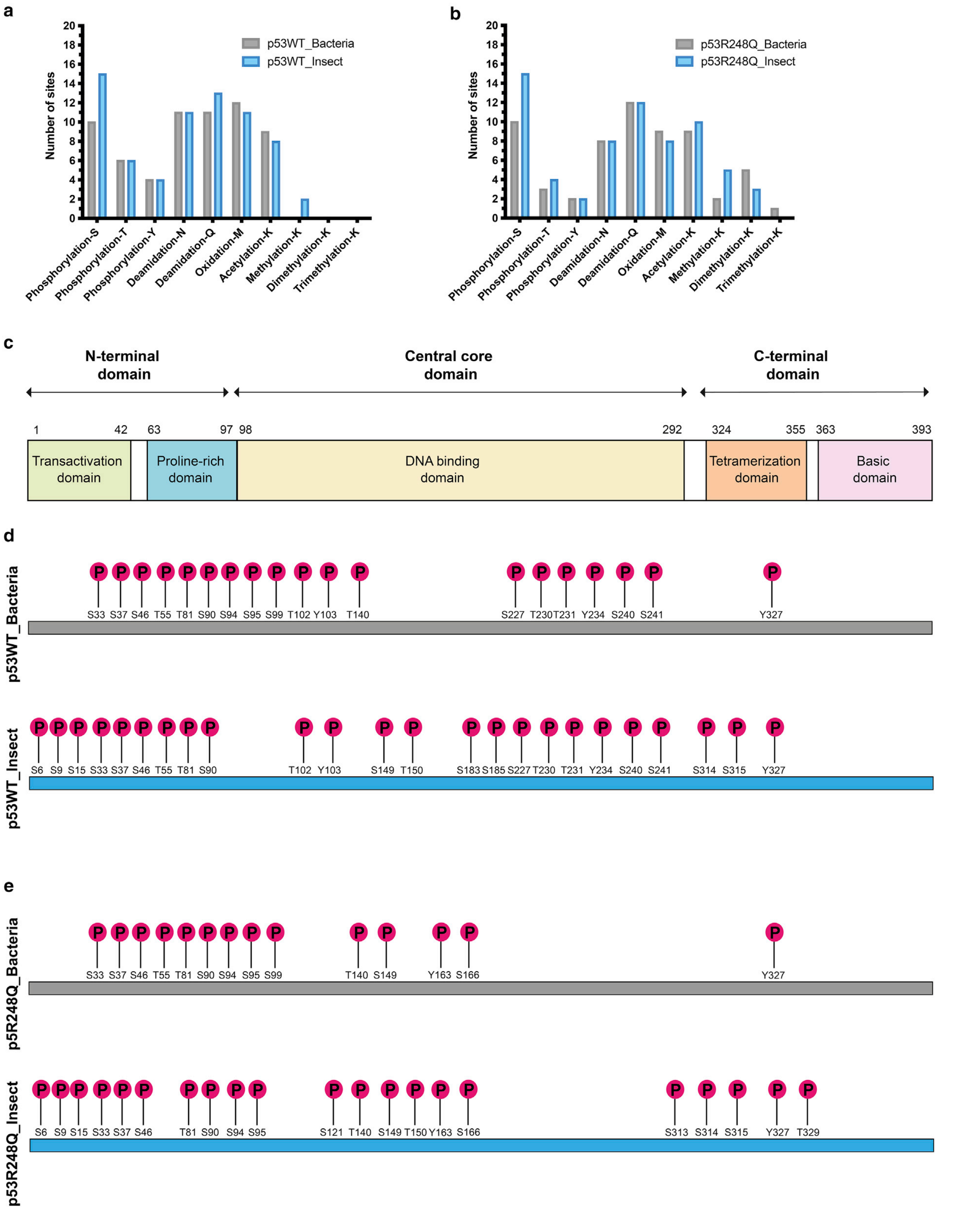

FIGURE 1 PTMs in bacterial and insect-derived p53WT and p53R248Q recombinant proteins. (*a* and *b*) Number of sites with PTMs on bacterial and insect-derived p53WT (*a*) and p53R248Q (*b*) proteins identified by mass spectrometry. The modifications are phosphorylation of serine (S), threonine (T), and tyrosine (Y), deamidation of asparagine (N) and glutamine (Q), oxidation of methionine (M), and acetylation and methylation of lysine (K). (*c*) Protein domain organization of human p53. (*d* and *e*) Distribution of phosphorylation sites on serine (S), threonine (T), and tyrosine (Y) identified by mass spectrometry in bacterial and insect-derived p53WT (*d*) and p53R248Q (*e*) proteins.

and S15 in the N-terminal domain and S314 and S315 in the C-terminal domain found in the human-derived protein was identified in both of the insect cell-derived proteins but not in the proteins produced in bacterial cells (Fig. 1 *c–e*). The majority of these sites are phosphorylated in response to various stress signals and result in p53 stabilization, nuclear accumulation, and biochemical activation in response to DNA damage (28). Previous studies have also reported a comparable pattern of p53 phosphoisoforms in proteins obtained from a baculovirus expression system and primary human cells (29). Owing to their comparable phosphorylation pattern with human p53, insect-derived p53 proteins were used for subsequent studies.

PTMs on p53 are cell type and tissue dependent and are also determined by genotype and stimuli-specific responses with approximately 60 phosphorylation sites having been identified previously (Fig. S1; Table S1). We identified a total of 31 phosphorylation sites in one or more of the recombinant proteins analyzed, partly due to certain peptides not being detected during mass spectrometry analysis. As yet, none of the nine tyrosine residues in p53 are known to be phosphorylated in the human-derived p53 protein, but we found some of the tyrosine residues in our recombinant proteins to be phosphorylated (Table S1). Other PTMs, including acetylation, deamidation, methylation, and oxidation, were also identified on bacterial and insect cell-derived p53 proteins (Figs. 1 *a*, *b*, and S1). A complete catalog of sites of PTM and whether they have been reported previously is detailed in Table S1.

The R248Q mutant forms larger and a greater number of amorphous aggregates compared with the wild-type protein.

Circular dichroism (CD) spectroscopy was used to check the secondary structure content of insect-derived full-length p53WT and p53R248Q proteins. CD spectra were similar between both proteins (Fig. S2), and deconvolution of the spectra with BeStSel (27) estimated similar secondary structure content in both proteins (Table S2). We then adapted diffraction-limited fluorescence imaging to visualize aggregation of p53WT and p53R248Q under physiological conditions (pH 7.2 and 37°C). The proteins were investigated at physiological concentrations of 50 and 100 nM (30), and at a high concentration of 1 μM. Aggregation in microfuge tubes could not be used for this study because the stickiness and large size of the aggregates formed required additional processing, such as sonication, to allow their detailed characterizations. Instead, aggregation reactions were carried out in chambered glass-bottomed slides, which allowed for real-time imaging of aggregate formation while minimizing external perturbations. The solutions were added to form a droplet that did not contact the sides of the chamber in order to avoid aggregates adhering to the chamber sides (Fig. 2 *a*). Solutions of the aggregating proteins were incubated with a benzothiazole salt, ThT, which, upon binding to amyloid aggregates, leads to an increase in fluorescence intensity by several orders of magnitude (31), thus allowing aggregate visualization. We found that 10 and 20 μM ThT had an inhibitory effect on aggregate formation (32) (Fig. S3 *a* and *b*) and so a lower concentration of 1 μM was chosen for all subsequent imaging studies, which allowed aggregate visualization without significantly affecting their formation (Fig. S3 *c*).

Measurements of overall ThT intensity and the size and number of aggregates for each FOV revealed that both p53WT and p53R248Q aggregate over time and that the ThT intensity was dependent on the initial total monomer concentration (Fig. 2 *b–d*). An initial p53WT concentration of 50 nM showed little aggregation but p53R248Q formed small aggregates within 24 h (Fig. 2 *b* and *g*). ThT intensity measurements at 100 nM concentration showed that p53R248Q aggregated faster and formed larger-sized and a greater number of aggregates compared with the wild-type protein (Fig. 2 *b* and *f*). At 1 μM concentration, small clusters of aggregates were detected within an hour for both proteins (Fig. 2 *b* and *e*), which, in the case of the mutant protein, grew into larger clusters (Fig. 2 *b* and *c*).

At the more physiological concentration of 100 nM, measurements of the number and apparent size of ThT-positive aggregates showed that the initial numbers of ThT-positive aggregates formed by both proteins were similar (Fig. 2 *h* and *i*). After 4 h, the number of p53R248Q aggregates grew rapidly over a period of 60 h compared with p53WT, with significant differences in aggregate numbers detected from 8 h onward (Fig. 2 *h*). The p53WT aggregates did not change significantly in size, and approximately 80% remained less than 100 μm^2 for over 3 days (Fig. 2 *i*). The p53R248Q aggregates were initially similar in size to the p53WT aggregates; however, they became progressively more heterogeneous in size (Fig. 2 *i*) and grew to greater than 300 μm^2, with the largest aggregates being approximately 3000 μm^2, whereas none of the p53WT aggregates were greater than 1000 μm^2. The increase in number and size of aggregates over time was not due to the presence of preformed seeds in the starting sample (Fig. S4).

The rate-determining step in wild-type and mutant p53 protein aggregation is the nucleation and growth of aggregates, not the formation of an aggregation-prone monomeric precursor

To perform kinetic analysis of the aggregation reaction a measure of the amount of aggregated material as a function of time needs to be obtained; for example, by relating the ThT signal to the concentration of aggregates formed. In order to do so, we determined the equilibrium concentrations of the remaining soluble protein after 72 h of aggregation by western blotting (Figs. 3 *a* and S5 *a*). We used 1 μM protein for aggregation since the remaining total monomer concentrations for 100 nM protein were below the western blot detection limit. We found that the soluble protein concentration was 700 $\pm$ 70 nM and 100 $\pm$ 40 nM for p53WT and

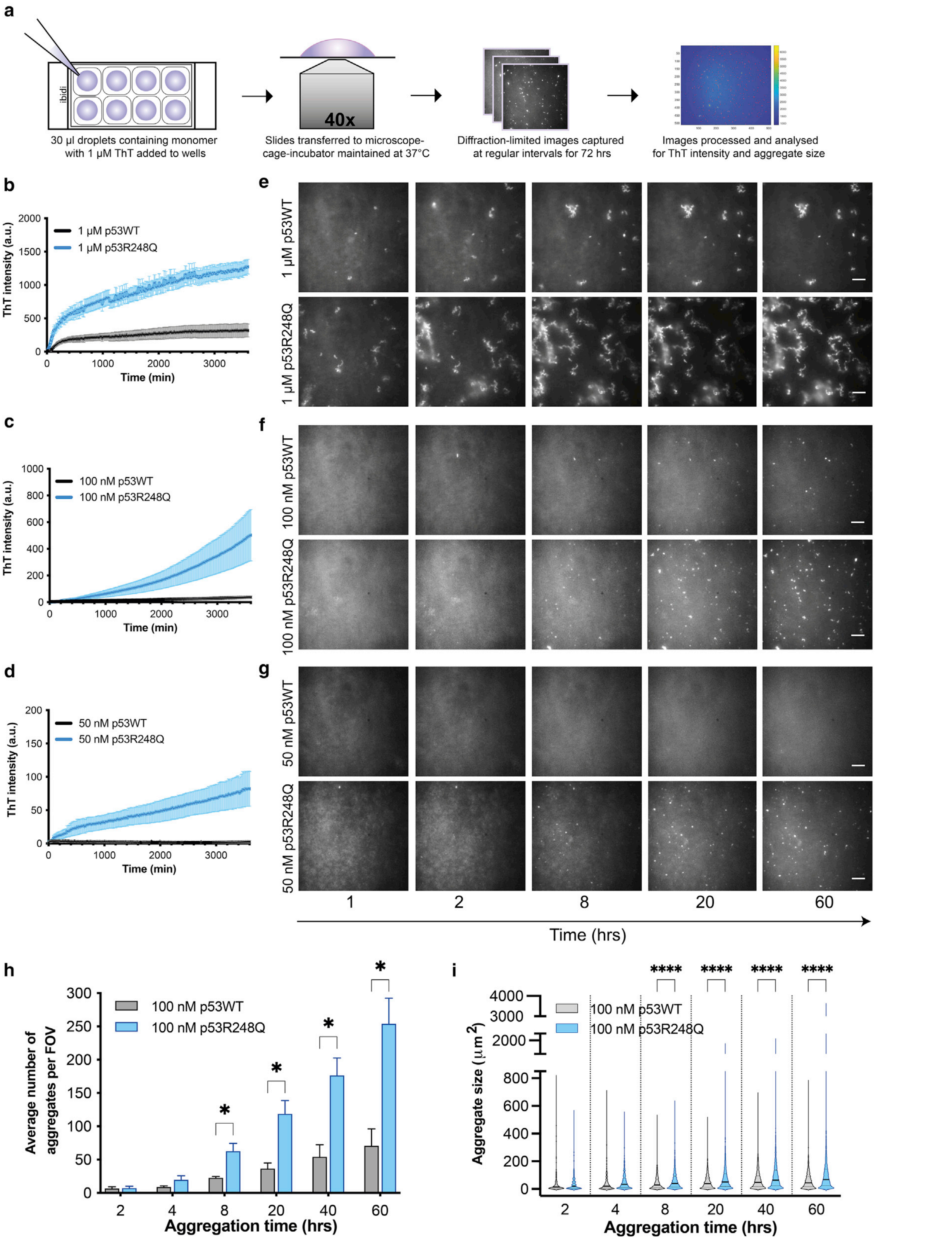

FIGURE 2 Kinetics of p53WT and p53R248Q aggregate formation imaged in real time using thioflavin T. (*a*) Schematic representation of droplet setup in chambered glass slides and diffraction-limited imaging for studying aggregation kinetics under physiological conditions, thus allowing quantitative analysis of the number and size of aggregates formed over time. (*b*–*d*) Measurements of aggregation from total ThT fluorescence intensity at different concentrations of 1 μM (*b*), 100 nM (*c*), and 50 nM (*d*) for p53WT and p53R248Q. All experiments were performed at 37°C, pH 7.2. Error bars represent standard errors of

(legend continued on next page)

p53R248Q, respectively, demonstrating a 90% reduction in total mutant monomer concentration compared with just 20% in p53WT at the end of 72 h of aggregation (Fig. 3 *b*). Notably, these concentrations of soluble species are higher than the total protein concentrations at which we observed small aggregated species by imaging. Such an effect has been observed previously in other systems such as α-synuclein and Aβ42 at low concentrations (33,34) and implies that the predominant structures formed at 100 nM are likely to be soluble oligomeric intermediates rather than more mature insoluble aggregates. This finding is in agreement with the distinct morphologies at 100 nM and 1 μM, observed by imaging. This in turn implies that the species detected at 1 μM may not be present at lower protein concentration, thus kinetic analysis was limited to the data obtained at 1 μM, to describe the formation of the more mature species.

Using the solubilities determined above, we were able to convert the observed ThT signal to aggregate concentrations and thus analyze aggregation kinetics quantitatively (35) (Fig. 3 *b*). The most crucial feature of these data is the initially increasing convex slope, which implies an increase in the rate of overall aggregate formation as the reaction proceeds. This is a hallmark of nucleated-growth, where the rate increases from its initial value as additional new aggregates are formed through primary nucleation. As nuclei accumulate, the growth rate increases, giving rise to upward curvature of the aggregation curves. This is in contrast to reactions limited purely by the availability of aggregation-prone precursors, in which the kinetic curves are expected to show no upward curvature. Such a situation can arise when nucleation and growth are very fast, as has been observed previously for other p53 constructs and at higher concentrations (19,20,36). The much slower speed of aggregation observed in our experiments is likely due to a combination of both the lower protein concentrations and the difference in the protein itself (full length instead of the core domain). Due to this decreased aggregation rate, the production of aggregation-prone species, for example due to an unfolding reaction, appears to no longer be the rate-limiting process, and thus the kinetics of the aggregation process itself can be sampled directly.

We therefore used a classical nucleation-growth model, where the differential equations describing the time evolution of the number of aggregates, P(t), and the concentration of aggregation-prone precursor, m(t) are:

$$\frac{dP}{dt} = k_n m(t)^{n_c}, \quad \frac{dm}{dt} = -2\left(k_+ m(t) - k_{off}\right) P(t) \qquad \text{(Equation 1)}$$

where k_+, k_{off} and k_n are the rates of growth, aggregate dissociation, and nucleation respectively, and n_c is the reaction order of primary nucleation. ThT fluorescence reports on the mass of aggregates, which is simply given by m(initial) − m(t).

In summary, the rates are:

$$\lambda(\mathrm{WT}) = 7e - 9s^{-2}$$

$$\lambda(\mathrm{WT}) = 2e - 8\,s^{-2}$$

$$k_{off}/k_+(\mathrm{WT}) = 7e - 7\mathrm{M}$$

$$k_{off}/k_+(\mathrm{mut}) = 1e - 7\mathrm{M}$$

where $\lambda = k_n k_+ \, m^{nc}$ is the combined nucleation-elongation rate and k_{off}/k_+ is the ratio of the dissociation rate constant to the elongation rate constant. Note that no assumptions about the nature of the monomeric precursor are needed here; i.e., the precursor may be a monomeric or a multimeric species, as the conclusions remain unchanged regardless. If, for example, the precursor was tetrameric, the concentrations would therefore be a factor of 4 lower than used above, meaning that the value of the parameters with units of concentration, i.e., the ratio k_{off}/k_+, would need to be adjusted accordingly. The relative changes between mutant and wild type discussed below would, however, remain unaffected.

The data are fit very well by this model, with one free parameter: the combined rate of elongation and nucleation. This combined rate is a factor of 3 faster for the mutant than the wild type. Additionally, the solubilities can be used to estimate the rates of dissociation relative to the growth rates. We find that the dissociation rate is likely decreased by between a factor of 3 and a factor of 7 for the mutant, relative to the wild type. The combined nucleation-elongation rate is comparable with the rate at which actin aggregates in vitro (37) and is significantly faster than other proteins that form pathological amyloids, such as Aβ (38,39) (Fig. S5 *b*). The high aggregation speed is consistent with the difficulty of finding experimental conditions under which it is the rate-limiting process. Notably, there is also no evidence of secondary nucleation mechanism for p53; i.e., a process by which existing aggregates catalyze the formation of new aggregates. By contrast, such processes dominate the aggregation of the majority of other disease-associated amyloids (39–42).

the mean from three or more independent experiments. Corresponding panels show representative diffraction-limited time-lapse images (*e–g*) of the aggregates formed. Scale bar, 10 μm. (*h*) Average number of aggregates per field of view (FOV) at indicated times during aggregation. Error bars represent standard error of the mean from three or more independent experiments (*$p < 0.05$). (*i*) Violin plots showing aggregate sizes at indicated times during aggregation. Solid lines represent the median and dotted lines represents the upper and lower quartiles (****$p < 0.0001$).

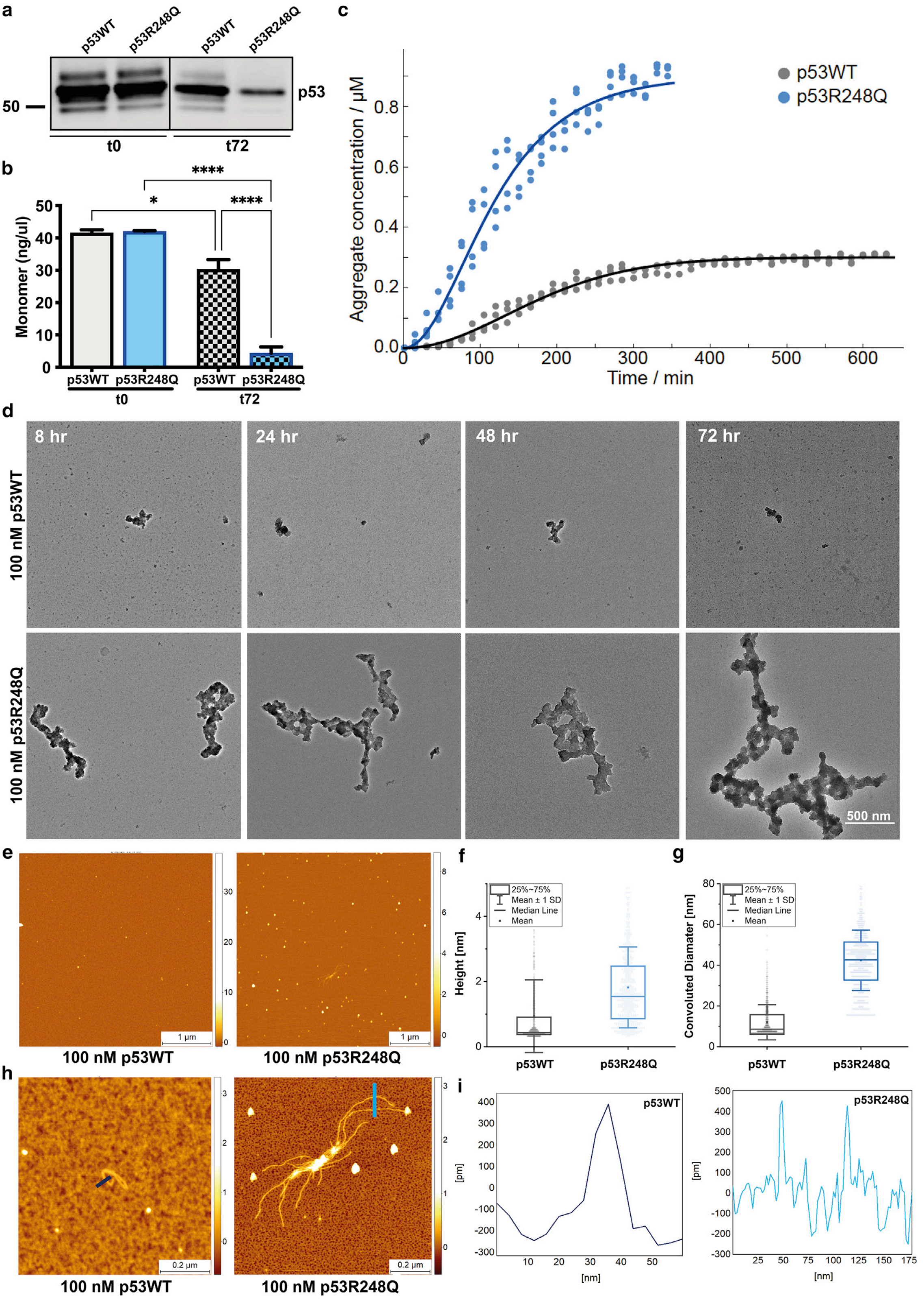

FIGURE 3 Modeling p53 aggregation kinetics and morphological characterization of aggregates formed. (*a*) Western blot showing the total amount of p53WT and p53R248Q monomer in the droplet before aggregation (t0) and after 72 h of aggregation (t72). (*b*) Concentration of total monomer in the droplet before (t0) and after 72 h of aggregation (t72) of 1 μM protein. Concentrations were determined from a standard curve generated using a dilution series containing known amounts of p53 protein. Error bars represents standard error of the mean from three independent experiments (*$p < 0.05$,

(legend continued on next page)

TEM and AFM showed the presence of amorphous aggregates with rare protofilaments

Protein solutions were aggregated directly on gold-coated TEM grids, and micrographs showed that aggregates formed by both p53WT and p53R248Q were generally amorphous and lacking the long, straight character of mature amyloid fibrils (43) (Fig. 3 *d*). Consistent with the ThT measurements, there was a significant size difference, with the majority of the p53WT aggregates being less than 400 nm across, whereas the p53R248Q aggregates grew into mesh-like structures that were approximately 1.5 μm across in 72 h. The aggregates remained amorphous over 3 days and exhibited no evidence of rearrangement into mature fibrils (Fig. 3 *d*).

We also exploited phase-controlled AFM high-resolution 3D imaging for morphological characterization of p53 aggregates. Proteins were aggregated at 37°C for 72 h and aliquots were collected and deposited onto positively functionalized mica substrates (44) to favor aggregate absorption. Imaging showed the presence of mostly spherical oligomeric species for both p53WT and p53R248Q (Fig. 3 *e*), consistent with the ThT and TEM studies, with the height and diameter of the mutant aggregates being almost four times larger than the wild-type counterparts (Fig. 3 *f* and *g*). Rare protofilaments were also observed in both samples (Fig. 3 *h*), which were larger and branched for p53R248Q. The protofilaments had a cross-sectional diameter of 0.4 nm, which corresponds to the width of a monomeric polypeptide chain (considering the protein molecular weight and hydrodynamic radius of p53) (Fig. 3 *i*).

Early p53WT and p53R248Q aggregates show a significant propensity to self-seed their aggregation

Seeds were generated by aggregating 100 nM samples in glass-bottomed wells for 8 h. The aggregates formed attached to the glass surface allowing the remaining protein to be removed by repeated washes (Figs. 4 *a* and S6 *a*). Similar number of p53WT and p53R248Q seeds were confirmed by imaging (Fig. S6 *b*) before freshly prepared 100 nM samples containing 1 μM ThT were added to wells containing preformed seeds. Homologous or self-seeded aggregation reactions included p53WT seeds with p53WT protein, and p53R248Q seeds with p53R248Q protein. Heterologous or cross-seeding reactions included p53WT seeds with p53R248Q, and p53R248Q seeds with p53WT protein. As a control, non-seeded protein solutions were also aggregated.

The capability of the wild-type and mutant pre-aggregated species to self- and cross-seed were significantly different (Fig. 4 *b*–*e*). Both p53WT (Fig. 4 *b* and *c*) and p53R248Q (Fig. 4 *b* and *e*) showed self-seeding, with a considerable increase in the initial rate of aggregation in the presence of the seeds. However, cross-seeding closely followed the aggregation kinetics of non-seeded aggregations for p53WT (Fig. 4 *b*) and there was only a minor increase in the rate of aggregation in the initial 24 h for cross-seeded p53R248Q (Fig. 4 *d*). The considerable acceleration of the aggregation rate in the presence of seeds contrasts with what has previously been observed (19). This constitutes further strong evidence that we are able to sample the aggregation itself, rather than formation of an aggregation-prone precursor.

Both p53WT and p53R248Q aggregates show susceptibility to Proteinase K digestion

The protease resistance property of amyloids and prions have been routinely utilized to characterize and biochemically separate infectious prions from their native cellular form (45). We examined the Proteinase K (PK) susceptibility of ThT-active p53 aggregates at different stages of aggregation by adding PK at different times to the glass surface on which the p53 aggregates were deposited. PK susceptibility was determined by measuring the fraction of ThT intensity of individual aggregates after 30 min of proteolytic digestion compared with that seen at the start of the measurement. A decrease in ThT intensity upon PK treatment would indicate susceptibility to PK (Fig. 5 *a*). Analysis of the remaining fraction of ThT intensity after 30 min in the absence of PK digestion showed that the ThT intensity in the majority of the aggregates obtained at different time points of aggregation for both p53WT (Fig. 5 *b*) and p53R248Q (Fig. 5 *a*) remained unchanged (value close to 1). However, there was a significant decrease in ThT intensity in aggregates of both p53WT (Fig. 5 *b*) and p53R248Q (Fig. 5 *c*) after 30 min of PK treatment, suggesting susceptibility to PK. Further analysis showed that there was no significant difference in PK susceptibility between very early (8-h aggregates) and late (72-h aggregates) stages of p53WT (Fig. 5 *d*) and p53R248Q (Fig. 5 *e*) aggregation, but a significant increase in partially sensitive species over time in both proteins. Density scatter plots of initial ThT intensity and remaining fraction of ThT intensity after 30 min of proteolytic treatment

****$p < 0.0001$). (*c*) Aggregation kinetic profiles of 1 μM p53WT and p53R248Q proteins, solid lines are fits of Eq. 1. (*d*) Representative TEM images of p53WT and p53R248Q were taken at the indicated time points during the course of aggregation. The 100 nM protein solutions were aggregated directly on gold-coated TEM grids. Scale bar, 500 nm. (*e*) Representative AFM morphology maps of p53WT and p53R248Q aggregates showing larger oligomeric species for p53R248Q. (*f* and *g*) Height and diameter of individual p53WT and p53R248Q spherical aggregates. (*h*) Rare protofilaments were observed in both p53WT and p53R248Q samples, but these were longer and branching with the R248Q mutation. (*i*) Cross-sectional height of protofilaments indicate that they are composed of a single chain of monomers.

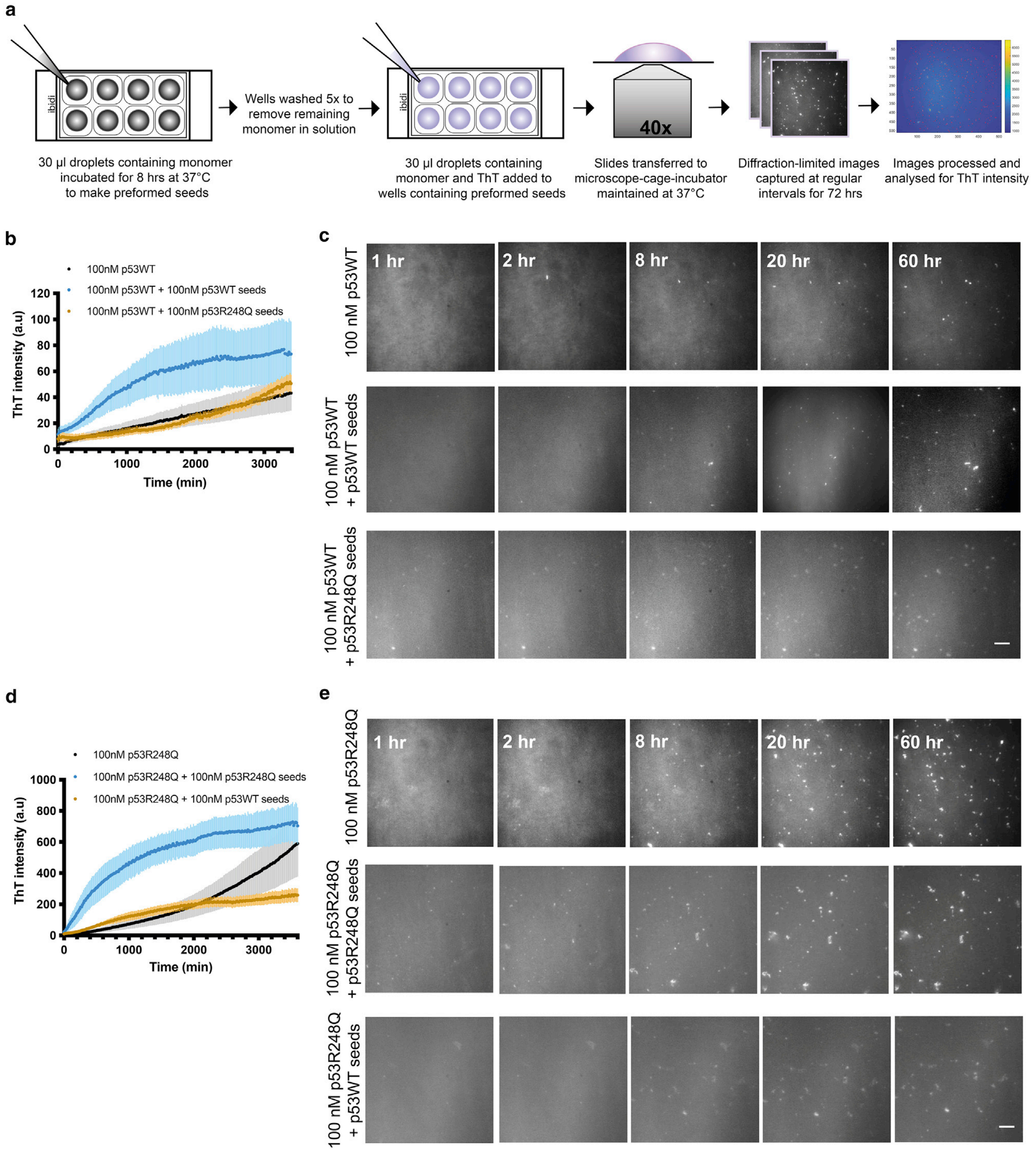

FIGURE 4 Kinetics of p53WT and p53R248Q seeded aggregation. (*a*) Schematic representation of the droplet setup in chambered glass slides and diffraction-limited imaging for studying seeded-aggregation kinetics under physiological conditions. (*b* and *d*) Measurements of aggregation from total ThT fluorescence intensity for 100 nM protein concentration(black), self-seeding (blue), and cross-seeding (orange) for p53WT (*b*) and p53R248Q (*d*). All experiments were performed at 37°C, pH 7.2. Error bars represent standard error of the mean of three or more independent experiments. (*c* and *e*) Corresponding panels showing representative diffraction-limited time-lapse images of aggregates formed during monomer-only and seeded aggregation. Scale bar, 10 μm.

show that the majority of the PK-sensitive aggregates for both p53WT and p53R248Q are the low-ThT-intensity (smaller) aggregates (Fig. S7). There is an increase in the population of higher-ThT-intensity aggregate species that are partially sensitive to PK (remaining fraction of ThT intensity between 0.5 and 0.7) in both p53WT and p53R248Q

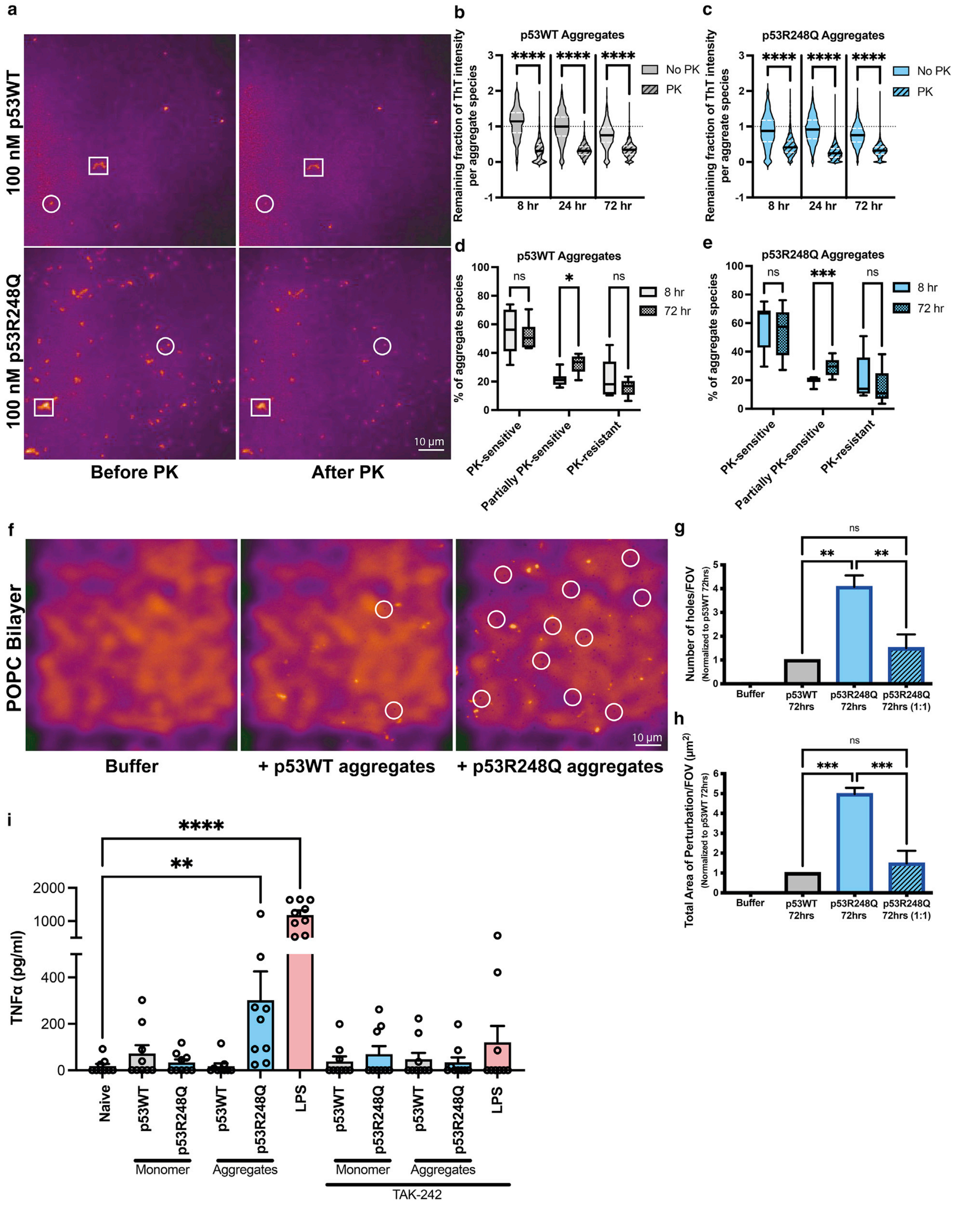

FIGURE 5 Functional characterization of p53 aggregates. (*a*) Representative diffraction-limited images of aggregates formed from 100 nM initial concentration showing ThT intensity of aggregates (orange) before and after Proteinase K (PK) treatment. PK sensitivity was determined by measuring the fraction of ThT intensity of individual aggregates after 30 min of proteolytic digestion compared with that seen at the start of the measurement. White squares show aggregates with a reduction in ThT intensity and white circles show aggregates with negligible ThT intensity after PK treatment. (*b* and *c*) Violin plots

(legend continued on next page)

over time (24- and 72-h aggregates), suggesting that larger aggregates formed by both over time share similar sensitivity to PK (Fig. S7).

Larger numbers of p53R248Q aggregates drive greater perturbation of a membrane bilayer preparation

Interactions of aggregates with cell membranes and the accompanying changes in membrane properties are considered to be one of the mechanisms responsible for the pathological consequences of amyloidosis (46). The consequences of this interaction are highly dependent on the nature of both the oligomers and the lipids. While the presence of anionic lipid vesicles has been shown to catalyze the aggregation of many amyloidogenic proteins ((47,48), aggregate-induced membrane permeabilization leading to cytotoxicity has also been reported (49). The ability of p53 aggregates to penetrate cells or induce cytotoxicity by compromising membrane integrity has been reported previously using aggregates formed by specific domains and/or at very high concentrations that may not be physiologically relevant (15,50,51).

To study the effect of physiological concentrations of full-length wild-type and mutant p53 aggregates on cell membranes, we utilized a supported lipid bilayer (SLB) where SUVs containing fluorescently labeled amphipathic lipids are layered onto a solid glass surface to form a lipid bilayer. Disruption of the bilayer is visible as holes in a fluorescence microscopy image obtained using TIRF illumination (Fig. 5 *f*). SLBs made from zwitterionic 1-palmitoyl-2-oleoyl-*sn*-glycero-3-phosphatidylcholine (POPC) were incubated with late-stage p53WT and p53R248Q aggregates prepared from 100 nM, which were sonicated prior to adding them to the membrane preparation. p53WT aggregates disrupted bilayer integrity within 24 h; however, the loss of integrity was four and five times more with p53R248Q aggregates, as measured by the number of holes formed (Fig. 5 *g*) and their total area (Fig. 5 *h*). Addition of BSA protein controls (Fig. S8) had no such disruptive effect on the lipid bilayer and was similar to buffer control (data not shown). The aggregates themselves became fluorescent after 24 h, suggesting that they had become coated by the fluorescent lipids released from the bilayer (Fig. 5 *f*). This suggests that both p53WT and p53R248Q aggregates can interact with a lipid bilayer and that the mutant aggregates exhibit a significantly more disruptive effect. The increased membrane disruption by p53R248Q aggregates could simply be a reflection of the higher number of aggregates formed compared with p53WT from an equivalent concentration. Decreasing the number of p53R248Q aggregates added to the bilayer to numbers similar to that observed with p53WT also reduced their disruptive effect of mutant aggregates, to levels similar to p53WT, suggesting that the greater disruption caused by p53R248Q aggregates was indeed driven by a larger number of aggregates and not necessarily by any inherent difference in aggregate properties.

p53R248Q aggregates invoke an inflammatory response in macrophages

Protein aggregates have been shown to interact with damage-associated molecular pattern receptors (DAMPS) such as TLR-4 on macrophages and induce an inflammatory response, which can be quantified by measuring the release of a pro-inflammatory cytokine, tumor necrosis factor alpha (TNFα) (52). Incubation of differentiated THP-1 human transformed macrophages with p53R248Q aggregates for 24 h resulted in significant release of TNFα, while p53WT aggregates did not invoke an inflammatory response (Fig. 5 *i*). The inflammatory response was only observed for early-stage (8 and 24 h) p53R248Q aggregates (Fig. S10), and late-stage (72 h) mutant aggregates induced negligible TNFα release (Fig. S10). Mutant p53R248Q aggregates formed in 24 h were able to induce significant TNFα release from macrophages compared with p53WT aggregates (Fig. 5 *i*). Non-aggregated controls were also included in the assay to ensure any macrophage activation was caused by the presence of aggregates themselves. The inflammatory response was blocked by pre-incubating the macrophages with TAK-242, which inhibits signaling via TLR-4 (53). These results could not be explained by the presence of endotoxin in the recombinant protein preparations, which were negligible at 0.006 and 0.003 pg of endotoxin per ng of p53WT and p53R248Q, respectively.

showing remaining fraction of ThT intensity in the absence or presence of PK for aggregates from different time points of aggregation (8, 24, 72 h) of p53WT (*b*) and p53R248Q (*c*). (*d* and *e*) Box-and-whisker plots show percentage of p53WT (*d*) and p53R248Q (*e*) aggregates that are PK sensitive (>70% loss in ThT intensity), partially PK sensitive (30%–70% loss in ThT intensity), or PK resistant (<30% loss in ThT intensity). The boxes represent the 25th to 75th percentiles and horizontal lines within the box represent median values. The whiskers represent the lowest and highest value (*$p < 0.05$, ***$p < 0.001$). (*f*) Representative images showing perturbation of POPC lipid bilayer upon addition of aggregates. Arrows indicate perturbation in the lipid bilayer. (*g* and *h*) Bar graphs showing number of holes formed in the bilayer per FOV (*g*) and their total area of perturbation per FOV (*h*) upon aggregate addition. The values are normalized to p53WT_72hrs. Mutant p53R248Q aggregates were also diluted 1:1 in buffer to reduce the number of aggregates added to the bilayer similar to the number of wild-type aggregates added. Error bars represent standard error of the mean of three or more independent experiments. (*i*) Inflammatory response determined by quantifying TNFα released by macrophages that had been incubated with non-aggregated samples or early-aggregate (24 h) samples for 24 h. The conditions were repeated on cells from the same differentiation that had been pre-incubated with the 1 μM TAK-242 for 1 h before co-incubating with the test preparations. Aggregates were prepared in three separate batches and each batch was assayed on three different Thp1 differentiations, which each had three technical replicates. Error bars represent standard error of mean.

DISCUSSION

Studying aggregation of insect-derived full-length p53 at sub-micromolar concentrations

Although protein aggregation and its role in disease pathogenesis has been confined mainly to neurodegenerative diseases, p53 aggregation has attracted interest in recent years, focusing on its involvement in cancer, given p53's crucial role as a tumor suppressor and master regulator of many cellular processes, such as the cell cycle, apoptosis, DNA repair, inflammation, and metabolism (5,14,51). This has led to several studies on p53 aggregation kinetics and the biophysical properties of the resulting aggregates, but these works have focused mainly on specific domains of the protein, in particular the central core domain, and to a lesser extent on aggregates formed by the transactivation and tetramerization domains (11–18). Aggregation of full-length p53 protein has only received limited attention (4,19,20). This is partly due to the fact that purification of full-length p53 has proved challenging due to its aggregation propensity and the high content of disordered regions (9). Hence, many aggregation studies reported previously have used the central core DNA-binding domain since it is structured, folds independently of the N- and C-terminal regions, and dictates the stability of the full-length protein (54).

To our knowledge, previous studies exploring p53 aggregation have been largely carried out using recombinant p53 produced in *E. coli*. Although bacterial expression systems continue to dominate the recombinant protein production field, largely due to cost considerations and ease of use, it is still not the preferred system for proteins that display PTMs. PTMs that influence protein structure and function may also alter their aggregation potential, as has been observed in several neurodegenerative proteinopathies (24). Regulation of p53 function is of vital importance for cell fate decisions, which is achieved through a myriad of processes, among which PTMs play critical roles (55). p53 stability, conformation, localization, and binding partners are regulated through PTMs, including phosphorylation, acetylation, methylation, and ubiquitination (55). Recombinant protein production in baculovirus-infected insect cells can produce large quantities of recombinant protein with PTMs resembling those of the native protein (29). Mass spectrometric analysis of full-length p53WT and mutant p53R248Q purified from bacterial and insect systems in this study identified a number of co-occurring and mutually exclusive PTMs, with the majority of differences in the phosphorylation of serines involved in p53 stabilization and activation in response to stress signals. Given the comparable phosphorylation patterns between p53 phosphoisoforms in proteins obtained from baculovirus expression systems and from primary human cells, as reported in an earlier study (29), and the presence of more phosphorylation sites in the insect-expressed p53 compared with the bacterial counterparts, we proceeded here to explore, under physiologically relevant conditions, the aggregation kinetics and biophysical properties of full-length insect-derived wild-type protein compared with one of the hotspot mutants in the DNA-binding domain, R248Q.

Previous studies have characterized aggregation of p53WT and several mutants at concentrations of 1–12 μM (4,19,20,22,56,57). In normal human cells with undamaged DNA, p53 concentration is so low that it is difficult to detect by conventional antibody-based techniques. The nuclear p53 concentration in exponentially growing normal human cells and tumorigenic cell lines calculated using the number of protein molecules per cell and assuming a nuclear diameter of 10 μm was found to be approximately 18–180 nM (30). Therefore, we limited our investigations to physiologically relevant concentrations of 100 nM or less, as well as a higher concentration of 1 μM. We coupled the specificity of ThT for aggregates with diffraction-limited fluorescence imaging to directly observe p53 aggregation in real time using glass-chambered slides. This is in contrast to conventional techniques for studying aggregation kinetics using standard plate-reader assays. Our method allowed us to obtain qualitative morphological information using TEM as well as quantitative data including aggregate size and numbers without the need for external perturbations, such as sonication.

Mechanism of p53 aggregation

Most amyloid-forming proteins aggregate by a nucleation-growth-type mechanism, where a slow nucleation process is followed by fast growth into larger aggregates. The aggregation of bacteria-derived recombinant p53 protein is believed to be limited mainly by the formation of an aggregation-prone precursor and has previously been modeled by a series of first-order reactions (36). By contrast, through our use of full-length, insect-derived p53 at low protein concentration, we were able to slow the aggregation process sufficiently for it to become the rate-limiting step in aggregate formation. This enabled fitting of the kinetics of aggregate formation at 1 μM to a simple nucleation-growth type model. The combined growth and nucleation rate is increased by a factor of 3 and the dissociation rate is decreased by a factor of between 3 and 7 for p53R248Q compared with p53WT. Thus, both the decreased solubility and increased aggregation rate could contribute to the increased aggregation propensity of the mutant protein. Previous work on dissociation constants of full-length p53 at 37°C (58) suggests that we could be working with a mixture of dimers and tetramers. However, this does not affect our kinetics analysis because, unlike in previous work on bacterial-derived p53 proteins where unfolding or dissociation of a tetramer is the rate-limiting step, nucleation and growth of aggregates are rate determining in our conditions.

When aggregate formation is rate limiting, introduction of even a small amount of preformed seed aggregates can

accelerate aggregation significantly. Our studies showed that the capability of the wild-type and mutant pre-aggregated species to self- and cross-seed were significantly different. Both p53WT and p53R248Q aggregates showed a significant propensity to self-seed but did not display a cross-seeding effect. These results thus clearly demonstrate that the actual aggregation of the protein, rather than just the production of an aggregation-prone precursor in an unimolecular reaction, limits the formation of aggregates. Thus, slowing aggregation itself, rather than slowing the formation of an aggregation-prone monomer, would be the most effective strategy to decrease the overall rate of aggregate formation in our systems. Unlike homologous seeding, not all amyloidogenic proteins are known to cross-seed, and the absence of conformational compatibility has been suggested as one of the main factors governing this (59). Our results contradict a previous report showing that bacterial-derived p53R248Q seeds can cross-seed p53WT (4). However, it should be noted that these studies had used high concentrations of seeds and monomer and the isolated core domains, which could influence nucleation.

Our results suggest that, in a cell, formation of soluble aggregates may occur at physiological concentrations of about 100 nM or even 50 nM p53WT. Since this has not been observed in normal cells carrying p53WT, it suggests that cellular aggregation of p53 is prevented by various protein quality-control mechanisms, including molecular chaperones. P53WT is a rapidly degraded protein and its cellular levels are tightly controlled by the proteosome-ubiquitin pathway. In response to different stresses, both wild-type and mutant p53 accumulate in cells (8). While p53WT rapidly returns to low basal levels following recovery from stress, mutant forms of p53 are stabilized and accumulate to high levels in cancer cells (60). If the missense R248Q mutation occurs, this will increase the rate of p53 aggregation by a factor of about 3–7. This is a significant increase and potentially, to a degree, may be sufficient to overcome the protective cellular mechanisms, which may already be dysregulated in cancer cells, thus leading to the accumulation of aggregates. Our data provide a plausible and simple mechanism of how the R248Q mutation can increase the aggregation propensity of p53.

The R248Q mutant forms a greater number of larger amorphous aggregates

The majority of the cancer-associated mutations in *TP53* are missense mutations, which are mostly clustered within the central DNA-binding domain region. One notable hotspot mp53 residue in cancer is R248, which is most frequently replaced by mutation with one of three amino acids, R248Q, R248W, and R248L, in many malignancies, including ovarian carcinoma, stomach and esophageal adenocarcinomas, gliomas, breast tumors, and lymphomas (61). The mutation has long been recognized as a DNA-contact mutant since R248 interacts directly with the minor groove of DNA (62). R248 is also one of the gatekeeper residues; i.e., charged residues that directly flank the APRs (residues 251–257 in p53) and that can slow aggregation (2). As reported previously (4), p53R248Q exhibited enhanced aggregate formation compared with p53WT. A recent study attributed the enhanced oligomerization of the R248Q mutant to allosteric changes in the N and C termini of the DNA-binding domain, thus exposing the APR at residues 251–257 (63). We found that the aggregation of p53R248Q was not just faster compared with the wild-type protein, it also formed larger sized and a greater number of aggregates.

The nature of p53 aggregates formed in vitro depends heavily on the aggregating conditions used, as shown by previous studies. Full-length protein or specific domains of wild-type and mutant p53 when subjected to certain conditions such as high pressure, high temperature, low pH, or RNA molecules, can form either amorphous or fibrillar aggregates (4,11,12,15,20,51,64–66). Although the best-studied examples of protein aggregation are those that form repeating fibrillar structures, we show that, under physiological temperature and pH, full-length p53WT and p53R248Q formed amorphous aggregates that were irregularly shaped and mesh-like, but still contained the β-structural elements responsible for ThT binding. AFM revealed the presence of mostly spherical aggregates but also the presence of rare protofilaments at late stages of aggregation with a cross section of 0.4 nm. Given the molecular weight of the monomer, this is consistent with a filament of diameter equivalent to one monomer, suggesting that the protofilaments are mostly monomeric chains, which is similar to what is observed in α-synuclein protofilaments (67). The majority of the oligomeric species for both proteins were spherical, with the diameter of the mutant aggregates being almost four times larger than the wild-type counterparts, which was consistent with the ThT and TEM data.

Toxicity of p53 aggregates

Protein aggregates, especially those implicated in neurodegenerative diseases, can be toxic to particular cell lines, either by direct disruption of cell membranes and subsequent ionic flux stress (68) or by stimulating an inflammatory response leading to cytokine toxicity and release of reactive oxygen species. Disruption of cell membranes would also support a mechanism in which a misfolded conformation is able to spread from one cell to another in a prion-like manner. Although full-length p53 has been shown to penetrate cancer cell lines and cause co-aggregation of intracellular p53 by macropinocytosis (50), prion-like spreading for p53 was demonstrated using amyloid seeds of a fragment of p53 (51). Our kinetic analysis of the formation of mature aggregates at 1 μM shows no evidence of the presence of an auto-catalytic fibril formation

mechanism, such as fibril fragmentation. Such a mechanism is present, for example, in Aβ42, which aggregates in Alzheimer's disease (39), and gives the aggregates the ability to self-replicate, as is required for true prions.

However, unlike the infectious forms of prions, which exhibit strong resistance to protease digestion (69), the majority of the p53WT and p53R248Q aggregates were sensitive to PK. These results are in agreement with our measurements of solubility with respect to the mature species determined above, which suggests that the aggregates formed at 100 nM are likely to be soluble oligomeric intermediates rather than more mature aggregates and are thus expected to be susceptible to PK digestion. We see an increase in partially sensitive species over time in both p53WT and p53R248Q, suggesting that they share similar PK sensitivity and possibly indicating that they may have similar structural rigidity. The higher fraction of partially PK-sensitive species at later time points could suggest a conformational change in both aggregate species over time. This conformational change could potentially contribute to changes in ThT binding affinity on the aggregate surface, as demonstrated by slight decrease in fraction of ThT intensity for 72-h wild-type and mutant aggregates in the absence of PK. However, the susceptibility of aggregates to protease treatment has been shown to be highly dependent on the environmental conditions in solution, especially the ionic strength and protease concentration (70). Hence, the biological relevance of this finding has to be carefully considered.

Despite the structural similarities between the aggregates formed, we see that p53R248Q aggregates showed a greater perturbation of a membrane bilayer preparation made from phosphatidylcholine compared with p53WT aggregates. This is likely a reflection of the greater number of aggregates formed by the mutant compared with the wild type from an equivalent concentration. We also showed that early p53R248Q aggregates can invoke an inflammatory response in macrophages, resulting in the release of the pro-inflammatory cytokine TNFα. This again could be because of the larger number of aggregates formed by the mutant protein and not necessarily by any inherent difference in aggregate properties. In contrast to the early-stage mutant aggregates, the late-stage p53R248Q aggregates failed to initiate an inflammatory response. This could possibly be due to their large size, which makes it difficult to interact with TLRs (71). It also remains to be shown whether membrane disruption resulting from p53 aggregation causes increased oxidative stress in cancer cells and/or results in their programmed cell death or necrosis. Additionally, it is also not clear whether this would inhibit tumor growth or lead to pathological inflammation and promotion of tumor growth. Although aggregated mutant p53 and different p53 isoforms have been reported in cancer cells and have been associated with loss-of-function, dominant-negative, or gain-of-function phenotypes (4,17,51,72–75), their exact role in tumorigenesis may be dependent on cell type and other factors that require further investigation.

CONCLUSIONS

Our results show that, at physiologically relevant concentrations, the R248Q mutation in the DNA-binding domain of p53 enhanced its aggregation propensity compared with the wild-type protein due to decreased solubility and increased aggregation rate, leading to the formation of a greater number of larger amorphous aggregates. Although there was no difference in sensitivity to protease digestion between wild-type and mutant p53 aggregates, mutant p53R248 aggregates were able to disrupt a lipid bilayer and provoke an inflammatory response. These findings suggest that, if enough aggregates are formed in vivo, they would be capable of altering the local environment around a cell with the R248Q p53 mutation. This could have important implications for cancer pathogenesis given the role inflammation plays in tumor initiation and progression.

DATA AVAILABILITY

All data and code are available upon request.

SUPPORTING MATERIAL

Supporting material can be found online at https://doi.org/10.1016/j.bpj.2022.10.013.

AUTHOR CONTRIBUTIONS

L.J., S.R., K.M.B., and D.K. conceived and designed the experiments. S.R. performed directed mutagenesis. L.J. performed bacterial expression and purification of p53 proteins. L.J. performed gel electrophoresis and data compilation for mass spectrometry experiments. L.J. and J.C.S. designed and performed the aggregation experiments (diffraction-limited imaging). L.J. performed soluble total monomer experiments. G.M. performed the kinetic analysis for the experimental data and interpreted the results. Y.W. and L.J. performed the TEM experiments. F.S.R. and A.M. performed the AFM experiments. L.J. and J.C.S. designed and carried out the Proteinase K experiments. Y.W. and M.R.C. performed the SLB experiments. J.H.B. and L.J. performed the inflammatory assay. L.J. and Y.W. performed the CD experiments. J.C.S. and Y.W. developed scripts for image analysis. S.R., K.M.B., and D.K. supervised the project. L.J. wrote the manuscript with input from all authors.

ACKNOWLEDGMENTS

We thank Cancer Research UK Cambridge Institute proteomics core facility manager Clive D'Santos and facility members Eva Papachristou and Valar Nila Roamio Franklin for the mass spectrometry experiment. We thank Alan Fersht's lab (MRC-Laboratory Molecular Biology) for providing bacterial plasmids for protein production. We acknowledge valuable conversations with members of both the Klenerman Lab at the Department of Chemistry and the Brindle Lab at Cancer Research UK Cambridge Institute. This work was funded by the CRUK Cambridge Centre Early Detection Programme Pump Priming Award to S.R. and D.K. (RG80668) and the

Multidisciplinary Project Award (26605). D.K. holds a Royal Society Professorship.

DECLARATION OF INTERESTS

The authors declare no competing interests.

REFERENCES

1. Chen, B., M. Retzlaff, …, J. Frydman. 2011. Cellular strategies of protein quality control. *Cold Spring Harbor Perspect. Biol.* 3:a004374.
2. De Baets, G., L. Van Doorn, …, J. Schymkowitz. 2015. Increased aggregation is more frequently associated to human disease-associated mutations than to neutral polymorphisms. *PLoS Comput. Biol.* 11:e1004374.
3. Xu, J., J. Reumers, …, J. Schymkowitz. 2011. Gain of function of mutant p53 by coaggregation with multiple tumor suppressors. *Nat. Chem. Biol.* 7:285–295.
4. Ano Bom, A. P. D., L. P. Rangel, …, J. L. Silva. 2012. Mutant p53 aggregates into prion-like amyloid oligomers and fibrils: implications for cancer. *J. Biol. Chem.* 287:28152–28162.
5. Levy, C. B., A. C. Stumbo, …, C. V. De Moura-Gallo. 2011. Co-localization of mutant p53 and amyloid-like protein aggregates in breast tumors. *Int. J. Biochem. Cell Biol.* 43:60–64.
6. Ostermeyer, A. G., E. Runko, …, U. M. Moll. 1996. Cytoplasmically sequestered wild-type p53 protein in neuroblastoma is relocated to the nucleus by a C-terminal peptide. *Proc. Natl. Acad. Sci. USA.* 93:15190–15194.
7. Hollstein, M., D. Sidransky, …, C. C. Harris. 1991. p53 mutations in human cancers. *Science.* 253:49–53.
8. Vousden, K. H., and C. Prives. 2009. Blinded by the light: the growing complexity of p53. *Cell.* 137:413–431.
9. Joerger, A. C., and A. R. Fersht. 2007. Structure-function-rescue: the diverse nature of common p53 cancer mutants. *Oncogene.* 26:2226–2242.
10. Bell, S., C. Klein, …, J. Buchner. 2002. p53 contains large unstructured regions in its native state. *J. Mol. Biol.* 322:917–927.
11. Lee, A. S., C. Galea, …, R. W. Kriwacki. 2003. Reversible amyloid formation by the p53 tetramerization domain and a cancer-associated mutant. *J. Mol. Biol.* 327:699–709.
12. Ishimaru, D., L. R. Andrade, …, J. L. Silva. 2003. Fibrillar aggregates of the tumor suppressor p53 core domain. *Biochemistry.* 42:9022–9027.
13. Butler, J. S., and S. N. Loh. 2003. Structure, function, and aggregation of the zinc-free form of the p53 DNA binding domain. *Biochemistry.* 42:2396–2403.
14. Higashimoto, Y., Y. Asanomi, …, K. Sakaguchi. 2006. Unfolding, aggregation, and amyloid formation by the tetramerization domain from mutant p53 associated with lung cancer. *Biochemistry.* 45:1608–1619.
15. Rigacci, S., M. Bucciantini, …, M. Stefani. 2008. The (1-63) region of the p53 transactivation domain aggregates in vitro into cytotoxic amyloid assemblies. *Biophys. J.* 94:3635–3646.
16. Pedrote, M. M., G. A. P. de Oliveira, …, J. L. Silva. 2018. Aggregation-primed molten globule conformers of the p53 core domain provide potential tools for studying p53C aggregation in cancer. *J. Biol. Chem.* 293:11374–11387.
17. Melo Dos Santos, N., G. A. P. de Oliveira, …, E. Rodrigues Pereira Gimba. 2019. Loss of the p53 transactivation domain results in high amyloid aggregation of the Delta40p53 isoform in endometrial carcinoma cells. *J. Biol. Chem.* 294:9430–9439.
18. Lima, I., A. Navalkar, …, E. A. Cino. 2020. Biophysical characterization of p53 core domain aggregates. *Biochem. J.* 477:111–120.
19. Wang, G., and A. R. Fersht. 2015. Propagation of aggregated p53: cross-reaction and coaggregation vs. seeding. *Proc. Natl. Acad. Sci. USA.* 112:2443–2448.
20. Wang, G., and A. R. Fersht. 2017. Multisite aggregation of p53 and implications for drug rescue. *Proc. Natl. Acad. Sci. USA.* 114:E2634–E2643.
21. Olivier, M., M. Hollstein, and P. Hainaut. 2010. TP53 mutations in human cancers: origins, consequences, and clinical use. *Cold Spring Harbor Perspect. Biol.* 2:a001008.
22. Wang, G., and A. R. Fersht. 2015. Mechanism of initiation of aggregation of p53 revealed by Phi-value analysis. *Proc. Natl. Acad. Sci. USA.* 112:2437–2442.
23. Sahdev, S., S. K. Khattar, and K. S. Saini. 2008. Production of active eukaryotic proteins through bacterial expression systems: a review of the existing biotechnology strategies. *Mol. Cell. Biochem.* 307:249–264.
24. Schaffert, L. N., and W. G. Carter. 2020. Do post-translational modifications influence protein aggregation in neurodegenerative diseases: a systematic review. *Brain Sci.* 10:E232.
25. Ruggeri, F. S., S. Vieweg, …, G. Dietler. 2016. Nanoscale studies link amyloid maturity with polyglutamine diseases onset. *Sci. Rep.* 6:31155.
26. Greenfield, N. J. 2006. Using circular dichroism spectra to estimate protein secondary structure. *Nat. Protoc.* 1:2876–2890.
27. Micsonai, A., F. Wien, …, J. Kardos. 2018. BeStSel: a web server for accurate protein secondary structure prediction and fold recognition from the circular dichroism spectra. *Nucleic Acids Res.* 46:W315–W322.
28. Bode, A. M., and Z. Dong. 2004. Post-translational modification of p53 in tumorigenesis. *Nat. Rev. Cancer.* 4:793–805.
29. Patterson, R. M., C. He, …, B. A. Merrick. 1996. Human p53 expressed in baculovirus-infected Sf9 cells displays a two-dimensional isoform pattern identical to wild-type p53 from human cells. *Arch. Biochem. Biophys.* 330:71–79.
30. Wang, Y. V., M. Wade, …, G. M. Wahl. 2007. Quantitative analyses reveal the importance of regulated Hdmx degradation for p53 activation. *Proc. Natl. Acad. Sci. USA.* 104:12365–12370.
31. LeVine, H., 3rd 1999. Quantification of beta-sheet amyloid fibril structures with thioflavin T. *Methods Enzymol.* 309:274–284.
32. Xue, C., T. Y. Lin, …, Z. Guo. 2017. Thioflavin T as an amyloid dye: fibril quantification, optimal concentration and effect on aggregation. *R. Soc. Open Sci.* 4:160696.
33. Novo, M., S. Freire, and W. Al-Soufi. 2018. Critical aggregation concentration for the formation of early Amyloid-beta (1-42) oligomers. *Sci. Rep.* 8:1783.
34. Iljina, M., G. A. Garcia, …, D. Klenerman. 2016. Kinetic model of the aggregation of alpha-synuclein provides insights into prion-like spreading. *Proc. Natl. Acad. Sci. USA.* 113:E1206–E1215.
35. Meisl, G., J. B. Kirkegaard, …, T. P. J. Knowles. 2016. Molecular mechanisms of protein aggregation from global fitting of kinetic models. *Nat. Protoc.* 11:252–272.
36. Wang, G., and A. R. Fersht. 2012. First-order rate-determining aggregation mechanism of p53 and its implications. *Proc. Natl. Acad. Sci. USA.* 109:13590–13595.
37. Higgs, H. N., L. Blanchoin, and T. D. Pollard. 1999. Influence of the C terminus of Wiskott-Aldrich syndrome protein (WASp) and the Arp2/3 complex on actin polymerization. *Biochemistry.* 38:15212–15222.
38. Meisl, G., X. Yang, …, T. P. J. Knowles. 2014. Differences in nucleation behavior underlie the contrasting aggregation kinetics of the Abeta40 and Abeta42 peptides. *Proc. Natl. Acad. Sci. USA.* 111:9384–9389.
39. Cohen, S. I. A., S. Linse, …, T. P. J. Knowles. 2013. Proliferation of amyloid-beta42 aggregates occurs through a secondary nucleation mechanism. *Proc. Natl. Acad. Sci. USA.* 110:9758–9763.

40. Padrick, S. B., and A. D. Miranker. 2002. Islet amyloid: phase partitioning and secondary nucleation are central to the mechanism of fibrillogenesis. *Biochemistry.* 41:4694–4703.
41. Gaspar, R., G. Meisl, …, S. Linse. 2017. Secondary nucleation of monomers on fibril surface dominates alpha-synuclein aggregation and provides autocatalytic amyloid amplification. *Q. Rev. Biophys.* 50:e6.
42. Meisl, G., C. K. Xu, …, T. P. J. Knowles. 2022. Uncovering the universality of self-replication in protein aggregation and its link to disease. *Sci. Adv.* 8:eabn6831.
43. Ke, P. C., R. Zhou, …, R. Mezzenga. 2020. Half a century of amyloids: past, present and future. *Chem. Soc. Rev.* 49:5473–5509.
44. Ruggeri, F. S., G. Longo, …, G. Dietler. 2015. Infrared nanospectroscopy characterization of oligomeric and fibrillar aggregates during amyloid formation. *Nat. Commun.* 6:7831.
45. Kushnirov, V. V., A. A. Dergalev, and A. I. Alexandrov. 2020. Proteinase K resistant cores of prions and amyloids. *Prion.* 14:11–19.
46. Gonzalez-Garcia, M., G. Fusco, and A. De Simone. 2021. Membrane interactions and toxicity by misfolded protein oligomers. *Front. Cell Dev. Biol.* 9:642623.
47. Lindberg, D. J., E. Wesén, …, E. K. Esbjörner. 2017. Lipid membranes catalyse the fibril formation of the amyloid-beta (1-42) peptide through lipid-fibril interactions that reinforce secondary pathways. *Biochim. Biophys. Acta Biomembr.* 1859:1921–1929.
48. Galvagnion, C., A. K. Buell, …, C. M. Dobson. 2015. Lipid vesicles trigger alpha-synuclein aggregation by stimulating primary nucleation. *Nat. Chem. Biol.* 11:229–234.
49. Andreasen, M., N. Lorenzen, and D. Otzen. 2015. Interactions between misfolded protein oligomers and membranes: a central topic in neurodegenerative diseases? *Biochim. Biophys. Acta.* 1848:1897–1907.
50. Forget, K. J., G. Tremblay, and X. Roucou. 2013. p53 Aggregates penetrate cells and induce the co-aggregation of intracellular p53. *PLoS One.* 8:e69242.
51. Ghosh, S., S. Salot, …, S. K. Maji. 2017. p53 amyloid formation leading to its loss of function: implications in cancer pathogenesis. *Cell Death Differ.* 24:1784–1798.
52. Hughes, C. D., M. L. Choi, …, D. Klenerman. 2019. Picomolar concentrations of oligomeric alpha-synuclein sensitizes TLR4 to play an initiating role in Parkinson's disease pathogenesis. *Acta Neuropathol.* 137:103–120.
53. Kawamoto, T., M. Ii, …, H. Kimura. 2008. TAK-242 selectively suppresses Toll-like receptor 4-signaling mediated by the intracellular domain. *Eur. J. Pharmacol.* 584:40–48.
54. Bullock, A. N., J. Henckel, …, A. R. Fersht. 1997. Thermodynamic stability of wild-type and mutant p53 core domain. *Proc. Natl. Acad. Sci. USA.* 94:14338–14342.
55. Liu, Y., O. Tavana, and W. Gu. 2019. p53 modifications: exquisite decorations of the powerful guardian. *J. Mol. Cell Biol.* 11:564–577.
56. Wilcken, R., G. Wang, …, A. R. Fersht. 2012. Kinetic mechanism of p53 oncogenic mutant aggregation and its inhibition. *Proc. Natl. Acad. Sci. USA.* 109:13584–13589.
57. Cino, E. A., I. N. Soares, …, J. L. Silva. 2016. Aggregation tendencies in the p53 family are modulated by backbone hydrogen bonds. *Sci. Rep.* 6:32535.
58. Rajagopalan, S., F. Huang, and A. R. Fersht. 2011. Single-Molecule characterization of oligomerization kinetics and equilibria of the tumor suppressor p53. *Nucleic Acids Res.* 39:2294–2303.
59. O'Nuallain, B., A. D. Williams, …, R. Wetzel. 2004. Seeding specificity in amyloid growth induced by heterologous fibrils. *J. Biol. Chem.* 279:17490–17499.
60. Sigal, A., and V. Rotter. 2000. Oncogenic mutations of the p53 tumor suppressor: the demons of the guardian of the genome. *Cancer Res.* 60:6788–6793.
61. Petitjean, A., E. Mathe, …, M. Olivier. 2007. Impact of mutant p53 functional properties on TP53 mutation patterns and tumor phenotype: lessons from recent developments in the IARC TP53 database. *Hum. Mutat.* 28:622–629.
62. Wong, K. B., B. S. DeDecker, …, A. R. Fersht. 1999. Hot-spot mutants of p53 core domain evince characteristic local structural changes. *Proc. Natl. Acad. Sci. USA.* 96:8438–8442.
63. Yang, D. S., A. Saeedi, …, P. G. Vekilov. 2021. Mesoscopic protein-rich clusters host the nucleation of mutant p53 amyloid fibrils. *Proc. Natl. Acad. Sci. USA.* 118:e2015618118.
64. DiGiammarino, E. L., A. S. Lee, …, R. W. Kriwacki. 2002. A novel mechanism of tumorigenesis involving pH-dependent destabilization of a mutant p53 tetramer. *Nat. Struct. Biol.* 9:12–16.
65. Kovachev, P. S., D. Banerjee, …, S. Sanyal. 2017. Distinct modulatory role of RNA in the aggregation of the tumor suppressor protein p53 core domain. *J. Biol. Chem.* 292:9345–9357.
66. Garg, A., J. P. Hazra, …, S. Sinha. 2020. Variable mutations at the p53-r273 oncogenic hotspot position leads to altered properties. *Biophys. J.* 118:720–728.
67. Ruggeri, F. S., F. Benedetti, …, G. Dietler. 2018. Identification and nanomechanical characterization of the fundamental single-strand protofilaments of amyloid alpha-synuclein fibrils. *Proc. Natl. Acad. Sci. USA.* 115:7230–7235.
68. Lee, S. J., P. Desplats, …, E. Masliah. 2010. Cell-to-cell transmission of non-prion protein aggregates. *Nat. Rev. Neurol.* 6:702–706.
69. Sajnani, G., and J. R. Requena. 2012. Prions, proteinase K and infectivity. *Prion.* 6:430–432.
70. Concha-Marambio, L., R. Diaz-Espinoza, and C. Soto. 2014. The extent of protease resistance of misfolded prion protein is highly dependent on the salt concentration. *J. Biol. Chem.* 289:3073–3079.
71. Sideris, D. I., J. S. H. Danial, …, D. Klenerman. 2021. Soluble amyloid beta-containing aggregates are present throughout the brain at early stages of Alzheimer's disease. *Brain Commun.* 3:fcab147.
72. Pedrote, M. M., M. F. Motta, …, G. A. P. de Oliveira. 2020. Oncogenic gain of function in glioblastoma is linked to mutant p53 amyloid oligomers. *iScience.* 23:100820.
73. Yang-Hartwich, Y., M. G. Soteras, …, G. Mor. 2015. p53 protein aggregation promotes platinum resistance in ovarian cancer. *Oncogene.* 34:3605–3616.
74. Silva, J. L., C. V. De Moura Gallo, …, L. P. Rangel. 2014. Prion-like aggregation of mutant p53 in cancer. *Trends Biochem. Sci.* 39:260–267.
75. Silva, J. L., E. A. Cino, …, G. A P de Oliveira. 2018. Targeting the prion-like aggregation of mutant p53 to combat cancer. *Acc. Chem. Res.* 51:181–190.

Biophysical Journal, Volume *121*

Supplemental information

Characterization of full-length p53 aggregates and their kinetics of formation

Linda Julian, Jason C. Sang, Yunzhao Wu, Georg Meisl, Jack H. Brelstaff, Alyssa Miller, Matthew R. Cheetham, Michele Vendruscolo, Tuomas P.J. Knowles, Francesco Simone Ruggeri, Clare Bryant, Susana Ros, Kevin M. Brindle, and David Klenerman

SUPPLEMENTARY INFORMATION

Characterization of full-length p53 aggregates and their kinetics of formation

Linda Julian[1,2], Jason C. Sang[2], Yunzhao Wu[2], Georg Meisl[2], Jack H. Brelstaff[3,4], Alyssa Miller[5], Matthew R. Cheetham[2], Michele Vendruscolo[5], Tuomas P. J. Knowles[5], Francesco Simone Ruggeri[5,6,7], Clare Bryant[4,8], Susana Ros[1], Kevin M. Brindle[1*], David Klenerman[2,9*]

Affiliations

1. Cancer Research UK Cambridge Institute, University of Cambridge, Cambridge, UK
2. Yusuf Hamied Department of Chemistry, University of Cambridge, Cambridge, UK
3. Department of Clinical Neurosciences, University of Cambridge, Cambridge, UK
4. Department of Medicine, University of Cambridge, Cambridge, UK
5. Centre for Misfolding Diseases, Yusuf Hamied Department of Chemistry, University of Cambridge, Cambridge, UK
6. Present Address: Laboratory of Organic Chemistry, Wageningen University & Research, Wageningen, Netherlands
7. Present Address: Laboratory of Physical Chemistry, Wageningen University & Research, Wageningen, Netherlands
8. Department of Veterinary Medicine, University of Cambridge, Cambridge, UK
9. UK Dementia Research Institute, University of Cambridge, Cambridge, UK

* Corresponding authors

Supplementary Figures

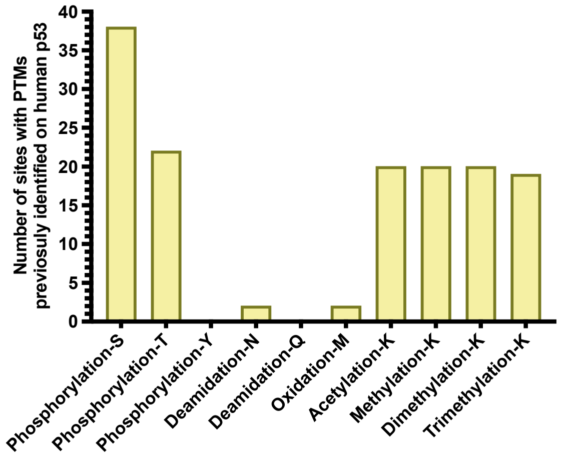

Figure S1. Post-translational modifications reported in human p53. Number of sites with post-translational modifications reported previously in human p53. The modifications include: phosphorylation of serine (S), threonine (T) and tyrosine (Y), deamidation of asparagine (N) and glutamine (Q), oxidation of methionine (M), acetylation and methylation of lysine (K).

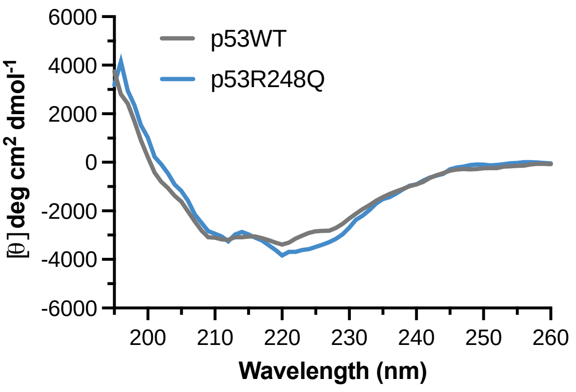

Figure S2. Circular dichroic (CD) spectra of p53WT and p53R248Q. CD spectra were recorded for insect-derived full-length p53WT and p53R248Q at a concentration of 2.5 µM and presented as a plot of molar ellipticity [θ] as a function of wavelength. Values were averaged from three accumulations of spectral scans, baseline corrected and the net spectrum was smoothed using the Savitsky-Golay method.

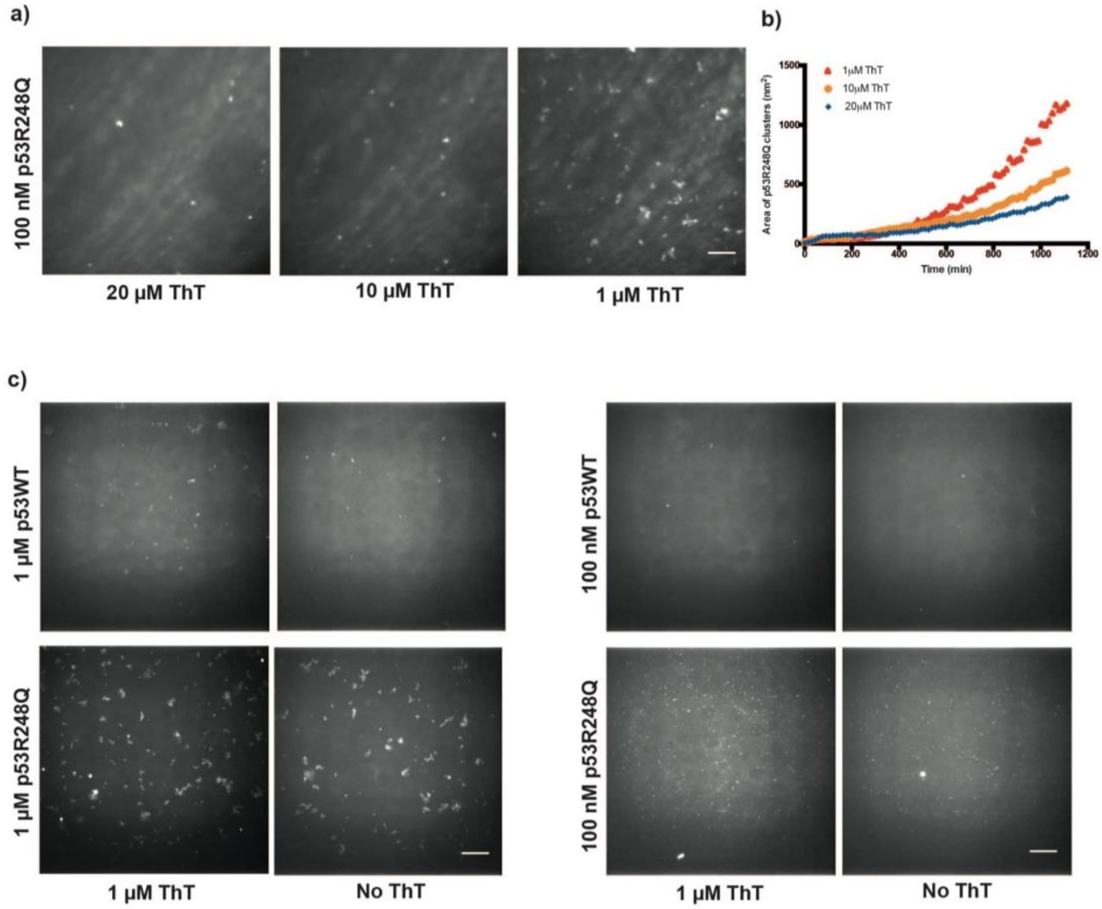

Figure S3. Effect of Thioflavin T (ThT) on p53 aggregation. (**a**) Representative images of insect-derived p53R248 aggregated for 18 hours with different concentrations of ThT. (**b**) Area occupied by p53R248 aggregate clusters as a function of time showing inhibitory effect of high concentration of ThT on aggregation. All experiments were performed in chambered glass slides at 37°C, pH 7.2. (**c**) Representative images of 1 µM and 100 nM p53WT and p53R248Q aggregated for 72 hours in the absence or presence of 1 µM ThT. Scale bar = 10 µm.

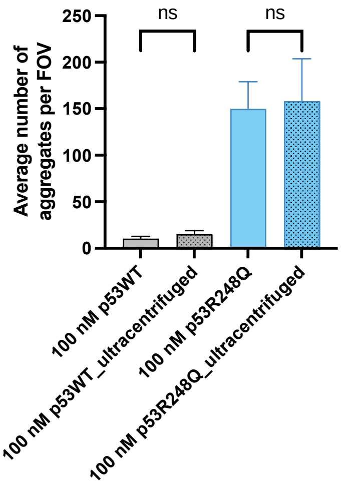

Figure S4. Absence of preformed seeds. Average number of aggregates per field of view (FOV) formed after 72 hours of aggregation of 100 nM p53WT and p53R248Q with and without prior ultracentrifugation. Error bars represents standard error of the mean (ns = not significant).

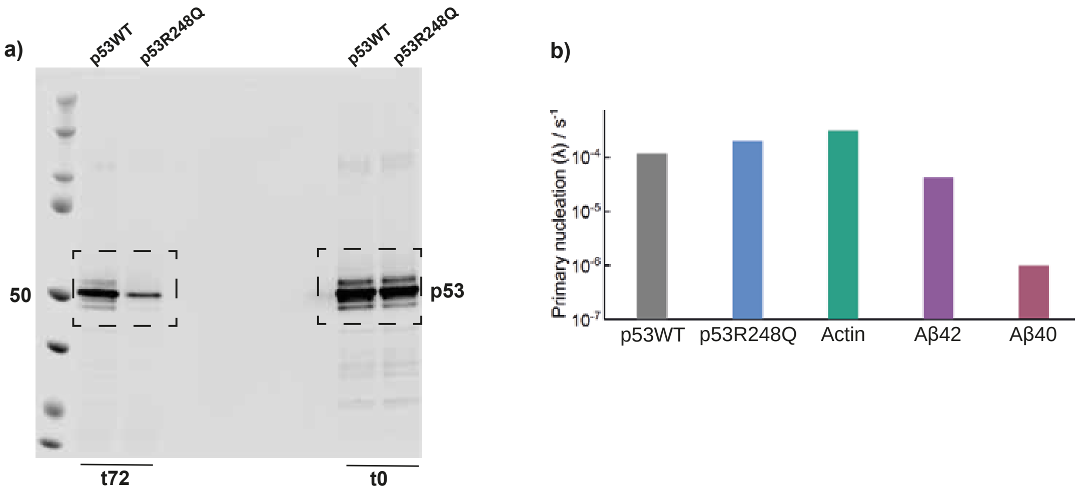

Figure S5. Modelling of p53 aggregation kinetics. (a) Uncropped western blot panels for Figure 3a, boxes with dashed border indicating regions used for the composite**.** (**b**) Bar graph showing primary nucleation rates of p53WT and p53R248Q compared to other aggregating proteins associated with neurodegenerative diseases using published rate constants and evaluated at a monomer concentration of 1 µM.

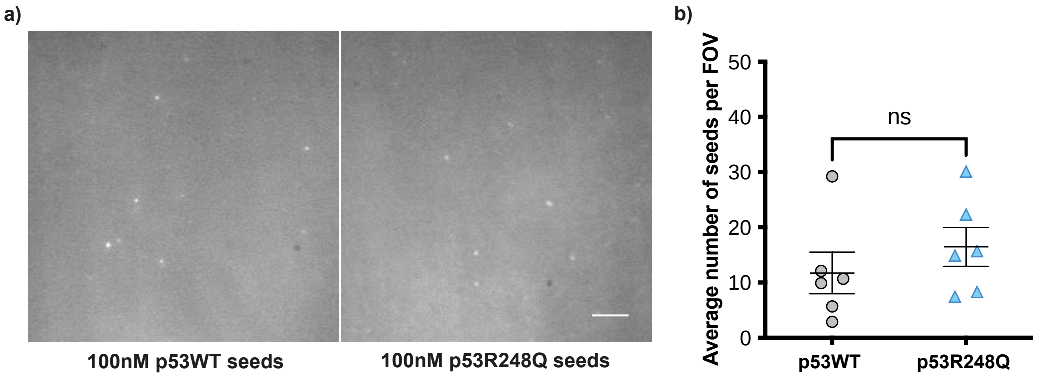

Figure S6. Generation of seeds. (**a**) Representative images showing presence of seeds on glass surface generated from aggregating 100 nM p53WT and p53R248Q for 8 hrs followed by repeated washes to remove remaining monomer. Scale bar = 10 µm. (**b**) Dot plot of average number of p53WT and p53R248Q seeds used for seeded aggregation reactions from 9 fields of view (FOV). Error bars represents standard error of the mean (ns = not significant).

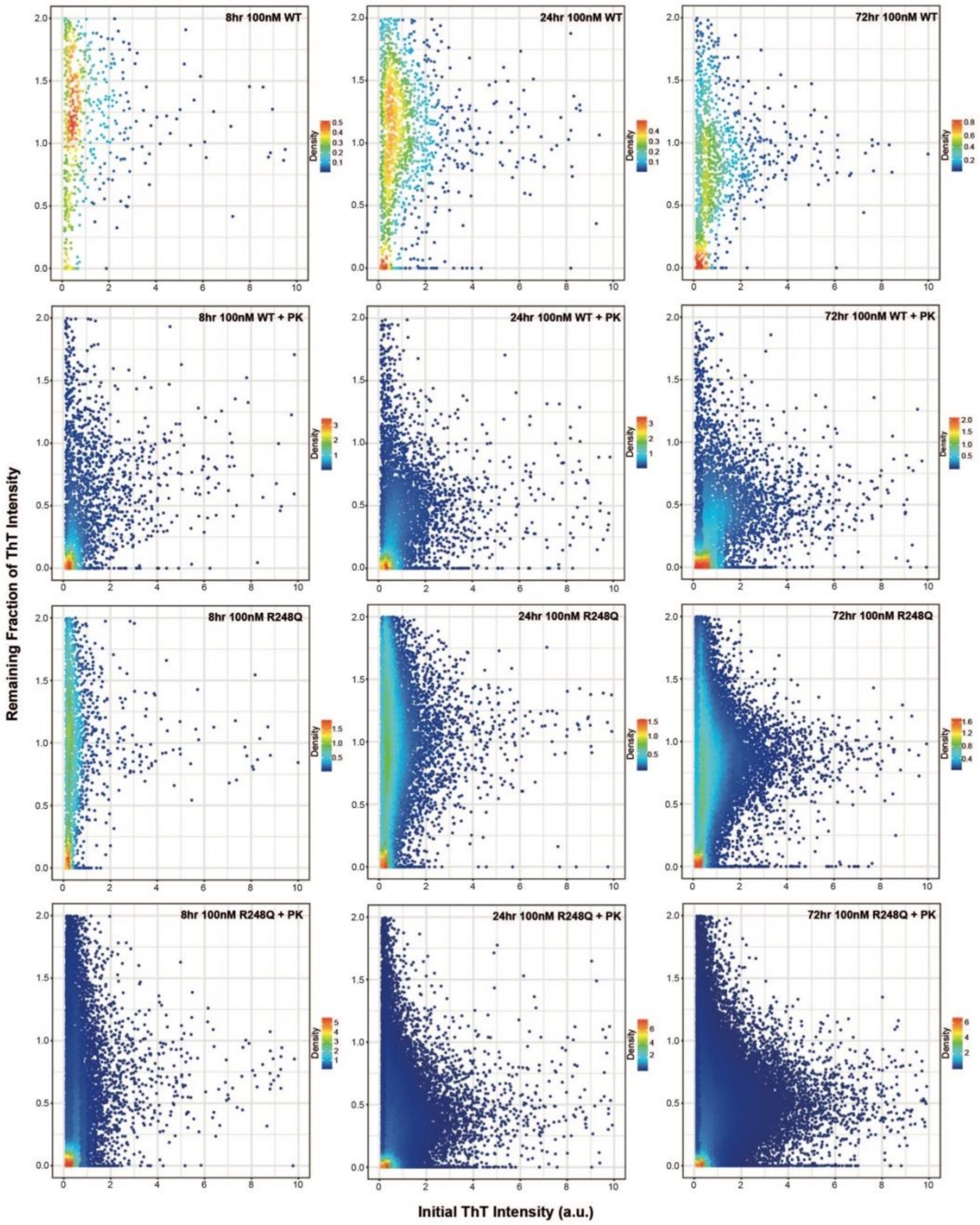

Figure S7. Sensitivity of p53 aggregates to Proteinase K (PK). Density scatter plots showing initial ThT intensity of aggregates of 100 nM p53WT and p53R248Q from different time points of aggregation (8, 24 and 72 hours) and the remaining fraction of ThT intensity after 30 minutes with or without proteolytic digestion.

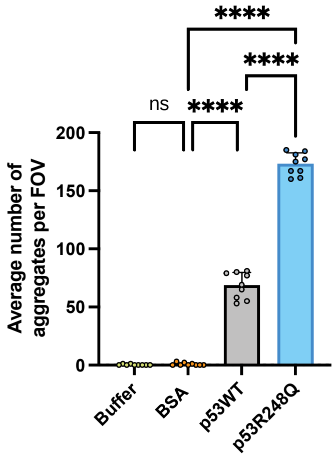

Figure S8. Number of aggregates added to the lipid bilayer. Average number of aggregates per field of view (FOV) formed after 72 hours of aggregation of equivalent concentrations of BSA, p53WT and p53R248Q. Error bars represents standard deviation (ns = not significant, **** P<0.0001).

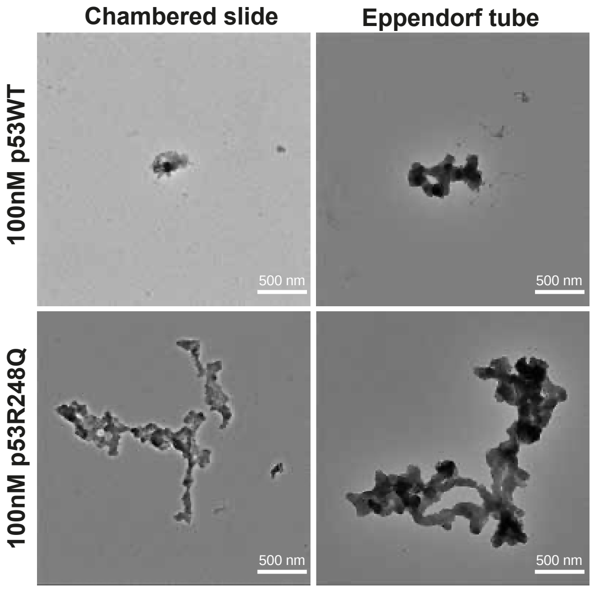

Figure S9. Amorphous aggregates. Representative TEM images of 100 nM p53WT and p53R248Q formed in chambered slides and in Eppendorf tubes show similar amorphous morphology and size differences. Scale bar = 500 nm

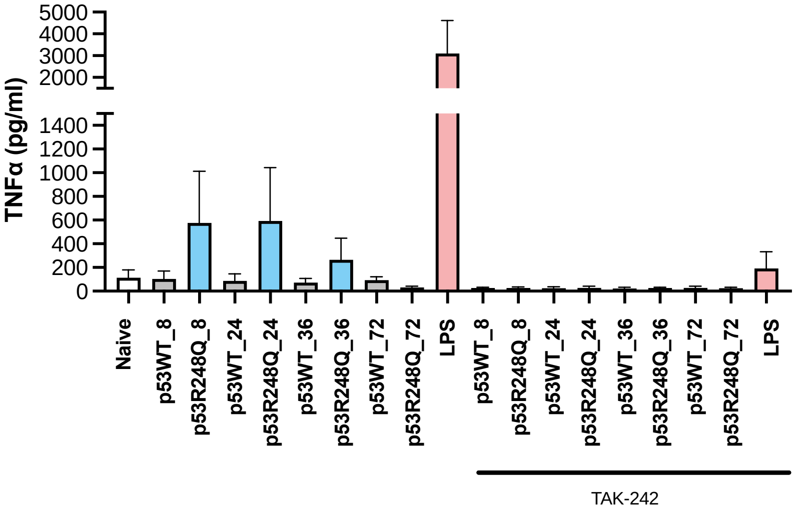

Figure S10. Inflammatory response of aggregates from different time points. Inflammatory response was determined by quantifying TNFα released by macrophages that had been incubated with aggregates (8, 24, 36, 72hr) samples for 24 hours. The conditions were repeated on cells from the same differentiation that had been pre-incubated with the 1 μM TAK-242 (TLR signalling inhibitor) for 1 hour before co-incubating with the test preparations for 24 hours. p53 aggregates were prepared in three separate batches and each batch was assayed on three different Thp1 differentiations which each had 3 technical replicates. Error bars represent standard error of mean.

Supplementary Tables

Table S1: Summary of post-translational modifications of asparagine (Asn), glutamine (Gln), lysine (Lys), methionine (Met), serine (Ser), threonine (Thr) and tyrosine (Tyr) residues found in full length wild-type and mutant p53 derived from bacterial and insect cells from mass spectrometry analysis. Residues within peptides that were not detected during mass spectrometry analysis are denoted as ND (Not Detected).

Residue	Modification	p53WT_ Bacteria	p53WT_ Insect	p53R248Q_ Bacteria	p53R248Q_ Insect	Status in Human cells	Reference
Met 1	Oxidation	✓	✓	✓	✓	Unknown	
Gln 5	Deamidation	✗	✓	✓	✓	Unknown	
Ser 6	Phosphorylation	✗	✓	✗	✓	Known	[1]
Ser 9	Phosphorylation	✗	✓	✗	✓	Known	[1]
Ser 15	Phosphorylation	✗	✓	✗	✓	Known	[2]
Gln 16	Deamidation	✗	✓	✗	✓	Unknown	
Thr 18	Phosphorylation	✗	✗	✗	✗	Known	[3]
Ser 20	Phosphorylation	✗	✗	✗	✗	Known	[1]
Lys 24	Acetylation	✗	✗	✗	✗	Known	[4]
	Methylation	✗	✗	✗	✗	Known	[4]
	Dimethylation	✗	✗	✗	✗	Known	[4]
Asn 29	Deamidation	✓	✓	✓	✓	Known	[5]
Asn 30	Deamidation	✓	✓	✓	✓	Known	[5]
Ser 33	Phosphorylation	✓	✓	✓	✓	Known	[1]
Ser 37	Phosphorylation	✓	✓	✓	✓	Known	[2]
Gln 38	Deamidation	✓	✓	✓	✓	Unknown	
Met 40	Oxidation	✓	✓	✓	✓	Known	[6]
Met 44	Oxidation	✓	✓	✓	✓	Unknown	
Ser 46	Phosphorylation	✓	✓	✓	✓	Known	[7]
Gln 52	Deamidation	✓	✓	✓	✓	Unknown	
Thr 55	Phosphorylation	✓	✓	✓	✗	Known	[8]
Met 66	Oxidation	✓	✓	✓	✓	Unknown	
Thr 81	Phosphorylation	✓	✓	✓	✓	Known	[9]
Ser 90	Phosphorylation	✓	✓	✓	✓	Known	[4]
Ser 94	Phosphorylation	✓	✗	✓	✓	Known	[4]
Ser 95	Phosphorylation	✓	✗	✓	✓	Known	[4]
Ser 96	Phosphorylation	✗	✗	✓	✗	Known	[4]
Ser 99	Phosphorylation	✓	✗	✓	✗	Known	[10]
Gln 100	Deamidation	✓	✓	✓	✓	Unknown	
Lys 101	Acetylation	✓	✓	✓	✓	Known	[4]
	Methylation	✗	✗	✗	✗	Known	[4]
	Dimethylation	✗	✗	✓	✗	Known	[4]

	Trimethylation	✗	✗	✗	✗	Known	[4]
Thr 102	Phosphorylation	✓	✓	✗	✗	Known	[4]
Tyr 103	Phosphorylation	✓	✓	✗	✗	Unknown	
Gln 104	Deamidation	✓	✓	✓	✓	Unknown	
Ser 106	Phosphorylation	✗	✗	✗	✗	Known	[11]
Ser 116	Phosphorylation	✗	✗	✗	✗	Known	[4]
Thr 118	Phosphorylation	✗	✗	✗	✗	Known	[4]
Lys 120	Acetylation	✓	✓	✓	✓	Known	[12]
	Methylation	✗	✗	✗	✗	Known	[4]
	Dimethylation	✗	✗	✗	✗	Known	[4]
	Trimethylation	✗	✗	✗	✗	Known	[4]
Ser 121	Phosphorylation	✗	✗	✗	✓	Known	[4]
Thr 123	Phosphorylation	✗	✗	✗	✗	Known	[4]
Thr 125	Phosphorylation	✗	✗	✗	✗	Known	[4]
Ser 127	Phosphorylation	✗	✗	✗	✗	Known	[4]
Asn 131	Deamidation	✓	✓	✓	✓	Unknown	
Lys 132	Acetylation	✓	✓	✓	✓	Known	[4]
	Methylation	✗	✗	✗	✗	Known	[4]
	Dimethylation	✗	✗	✗	✗	Known	[4]
	Trimethylation	✗	✗	✗	✗	Known	[4]
Met 133	Oxidation	✓	✓	✓	✓	Unknown	
Gln 136	Deamidation	✓	✓	✓	✓	Unknown	
Lys 139	Acetylation	✓	✓	✓	✓	Known	[4]
	Methylation	✗	✗	✗	✗	Known	[4]
	Dimethylation	✗	✗	✗	✗	Known	[4]
	Trimethylation	✗	✗	✗	✗	Known	[4]
Thr 140	Phosphorylation	✓	✗	✓	✓	Known	[4]
Gln 144	Deamidation	✓	✓	✓	✓	Unknown	
Ser 149	Phosphorylation	✗	✓	✓	✓	Known	[13]
Thr 150	Phosphorylation	✗	✓	✗	✓	Known	[13]
Thr 155	Phosphorylation	✗	✗	✗	✗	Known	[13]
Met 160	Oxidation	✓	✓	✓	✓	Known	[6]
Met 169	Oxidation	✓	✓	✓	✓	Unknown	
Tyr 163	Phosphorylation	✗	✗	✓	✓	Unknown	
Lys 164	Acetylation	✓	✓	✓	✓	Known	[14]
	Methylation	✗	✗	✗	✗	Known	[4]
	Dimethylation	✗	✗	✓	✗	Known	[4]
	Trimethylation	✗	✗	✗	✗	Known	[4]
Gln 165	Deamidation	✓	✓	✓	✓	Unknown	
Ser 166	Phosphorylation	✗	✗	✓	✓	Known	[4]
Gln 167	Deamidation	✓	✓	✓	✓	Unknown	
Thr 170	Phosphorylation	✗	✗	✗	✗	Known	[4]

Ser 183	Phosphorylation	✗	✓	✗	✗	Known	[15]
Ser185	Phosphorylation	✗	✓	✗	✗	Known	[4]
Gln 192	Deamidation	✓	✓	✓	✗	Unknown	
Asn 200	Deamidation	✓	✓	✓	✓	Unknown	
Asn 210	Deamidation	✓	✓	✓	✓	Unknown	
Thr 211	Phosphorylation	✗	✗	✗	✗	Known	[16]
Ser 215	Phosphorylation	✗	✗	✗	✗	Known	[17]
Ser 227	Phosphorylation	✓	✓	ND	ND	Known	[4]
Thr 230	Phosphorylation	✓	✓	ND	ND	Known	[4]
Thr 231	Phosphorylation	✓	✓	ND	ND	Known	[4]
Tyr 234	Phosphorylation	✓	✓	ND	ND	Unknown	
Asn 235	Deamidation	✓	✓	ND	ND	Unknown	
Tyr 236	Phosphorylation	✓	✓	ND	ND	Unknown	
Met 237	Oxidation	✓	✓	ND	ND	Unknown	
Asn 239	Deamidation	✓	✓	ND	ND	Unknown	
Ser 240	Phosphorylation	✓	✓	ND	ND	Known	[4]
Ser 241	Phosphorylation	✓	✓	ND	ND	Known	[4]
Met 243	Oxidation	✓	✓	ND	ND	Unknown	
Met 246	Oxidation	✓	✓	ND	ND	Unknown	
Asn 247	Deamidation	✓	✓	ND	ND	Unknown	
Thr 253	Phosphorylation	✗	✗	✗	✗	Known	[4]
Thr 256	Phosphorylation	✗	✗	✗	✗	Known	[4]
Ser 260	Phosphorylation	✗	✗	✗	✗	Known	[4]
Ser 261	Phosphorylation	✗	✗	✗	✗	Known	[4]
Ser 269	Phosphorylation	✗	✗	✗	✗	Known	[4]
Thr 284	Phosphorylation	✗	✗	✗	✗	Known	[15]
Lys 291	Acetylation	✗	✗	✗	✗	Known	[4]
	Methylation	✗	✓	✗	✗	Known	[4]
	Dimethylation	✗	✗	✗	✗	Known	[4]
	Trimethylation	✗	✗	✗	✗	Known	[4]
Lys 292	Acetylation	✗	✓	✓	✓	Known	[18]
	Methylation	✗	✓	✓	✓	Known	[4]
	Dimethylation	✗	✗	✗	✗	Known	[4]
	Trimethylation	✗	✗	✗	✗	Known	[4]
Ser 303	Phosphorylation	✗	✗	✗	✗	Known	[4]
Thr 304	Phosphorylation	✗	✗	✗	✗	Known	[4]
Lys 305	Acetylation	✗	✗	✗	✗	Known	[18]
	Methylation	✗	✗	✗	✗	Known	[4]
	Dimethylation	✗	✗	✓	✗	Known	[4]
	Trimethylation	✗	✗	✗	✗	Known	[4]
Asn 310	Deamidation	✓	✓	✓	✓	Unknown	
Asn 311	Deamidation	✓	✓	✓	✓	Unknown	

Thr 312	Phosphorylation	✗	✗	✗	✗	Known	[19]
Ser 313	Phosphorylation	✗	✗	✗	✓	Known	[19]
Ser 314	Phosphorylation	✗	✓	✗	✓	Known	[19]
Ser 315	Phosphorylation	✗	✓	✗	✓	Known	[2]
Gln 317	Deamidation	✓	✓	✓	✓	Unknown	
Lys 319	Acetylation	✓	✗	✓	✓	Known	[20]
	Methylation	✗	✗	✗	✗	Known	[4]
	Dimethylation	✗	✗	✗	✗	Known	[4]
	Trimethylation	✗	✗	✗	✗	Known	[4]
Lys 320	Acetylation	✓	✗	✗	✗	Known	[21]
	Methylation	✗	✗	✓	✓	Known	[4]
	Dimethylation	✗	✗	✗	✗	Known	[4]
	Trimethylation	✗	✗	✗	✗	Known	[4]
Lys 321	Acetylation	✓	✓	✓	✓	Known	[4]
	Methylation	✗	✗	✓	✓	Known	[4]
	Dimethylation	✗	✗	✓	✓	Known	[4]
	Trimethylation	✗	✗	✗	✗	Known	[4]
Tyr 327	Phosphorylation	✓	✓	✓	✓	Unknown	
Thr 329	Phosphorylation	✗	✗	✗	✓	Known	[4]
Met 340	Oxidation	✓	✓	✓	✓	Unknown	
Asn 345	Deamidation	✓	✓	✓	✓	Unknown	
Lys 351	Acetylation	✓	✓	✗	✓	Known	[4]
	Methylation	✗	✗	✗	✓	Known	[4]
	Dimethylation	✗	✗	✓	✓	Known	[4]
	Trimethylation	✗	✗	✓	✗	Known	[4]
Gln 354	Deamidation	✓	✓	✓	✓	Unknown	
Lys 357	Acetylation	✗	✗	✓	✓	Known	[22]
	Methylation	✗	✗	✗	✓	Known	[4]
	Dimethylation	✗	✗	✗	✓	Known	[4]
	Trimethylation	✗	✗	✗	✗	Known	[4]
Ser 362	Phosphorylation	✗	✗	✗	✗	Known	[23]
Ser 366	Phosphorylation	✗	✗	✗	✗	Known	[24]
Ser 367	Phosphorylation	✗	✗	✗	✗	Known	[4]
Lys 370	Acetylation	ND	ND	ND	ND	Known	[22]
	Methylation	ND	ND	ND	ND	Known	[25]
	Dimethylation	ND	ND	ND	ND	Known	[25]
	Trimethylation	ND	ND	ND	ND	Known	[25]
Ser 371	Phosphorylation	✗	✗	✗	✗	Known	[26]
Lys 372	Acetylation	ND	ND	ND	ND	Known	[27]
	Methylation	ND	ND	ND	ND	Known	[28]
	Dimethylation	ND	ND	ND	ND	Known	[29]
	Trimethylation	ND	ND	ND	ND	Known	[4]

Lys 373	Acetylation	ND	ND	ND	ND	Known	[27]
	Methylation	ND	ND	ND	ND	Known	[30]
	Dimethylation	ND	ND	ND	ND	Known	[30]
	Trimethylation	ND	ND	ND	ND	Known	[4]
Ser 376	Phosphorylation	✗	✗	✗	✗	Known	[26]
Thr 377	Phosphorylation	✗	✗	✗	✗	Known	[26]
Ser 378	Phosphorylation	✗	✗	✗	✗	Known	[26]
Lys 381	Acetylation	ND	ND	ND	ND	Known	[27]
	Methylation	ND	ND	ND	ND	Known	[4]
	Dimethylation	ND	ND	ND	ND	Known	[4]
	Trimethylation	ND	ND	ND	ND	Known	[4]
Lys 382	Acetylation	✗	ND	ND	ND	Known	[22]
	Methylation	✗	ND	ND	ND	Known	[31]
	Dimethylation	✗	ND	ND	ND	Known	[32]
	Trimethylation	✗	ND	ND	ND	Known	[4]
Met 384	Oxidation	✓	ND	✓	ND	Unknown	
Lys 386	Acetylation	✗	ND	✗	ND	Known	[33]
	Methylation	✗	ND	✗	ND	Known	[32]
	Dimethylation	✗	ND	✗	ND	Known	[32]
	Trimethylation	✗	ND	✗	ND	Known	[4]
Thr 387	Phosphorylation	✗	✗	✗	✗	Known	[24]
Ser 392	Phosphorylation	✗	✗	✗	✗	Known	[34]

Table S2. BeStSel secondary structure estimation of p53WT and p53R248Q protein from circular dichroism spectra.

**Secondary Structure**		**p53WT (%)**	**p53R248Q (%)**
**Helix**	Helix (regular)	4.1	4.5
	Helix (distorted)	3.7	3.9
**Antiparallel**	Anti (left-twisted)	1.6	5.3
	Anti (relaxed)	16.6	15.2
	Anti (right-twisted)	13.5	12.9
**Parallel**		0	0
**Turn**		13.7	14.1
**Others**		46.8	44.1

Supplementary References

1. Saito, S., et al., *Phosphorylation site interdependence of human p53 post-translational modifications in response to stress.* J Biol Chem, 2003. **278**(39): p. 37536-44.
2. Lees-Miller, S.P., et al., *Human DNA-activated protein kinase phosphorylates serines 15 and 37 in the amino-terminal transactivation domain of human p53.* Mol Cell Biol, 1992. **12**(11): p. 5041-9.
3. Sakaguchi, K., et al., *Damage-mediated phosphorylation of human p53 threonine 18 through a cascade mediated by a casein 1-like kinase. Effect on Mdm2 binding.* J Biol Chem, 2000. **275**(13): p. 9278-83.
4. DeHart, C.J., et al., *Extensive post-translational modification of active and inactivated forms of endogenous p53.* Mol Cell Proteomics, 2014. **13**(1): p. 1-17.
5. Lee, J.C., et al., *Protein L-isoaspartyl methyltransferase regulates p53 activity.* Nat Commun, 2012. **3**: p. 927.
6. Lu, J., et al., *MICAL2 Mediates p53 Ubiquitin Degradation through Oxidating p53 Methionine 40 and 160 and Promotes Colorectal Cancer Malignance.* Theranostics, 2018. **8**(19): p. 5289-5306.
7. Bulavin, D.V., et al., *Phosphorylation of human p53 by p38 kinase coordinates N-terminal phosphorylation and apoptosis in response to UV radiation.* EMBO J, 1999. **18**(23): p. 6845-54.
8. Gatti, A., et al., *Phosphorylation of human p53 on Thr-55.* Biochemistry, 2000. **39**(32): p. 9837-42.
9. Buschmann, T., et al., *Jun NH2-terminal kinase phosphorylation of p53 on Thr-81 is important for p53 stabilization and transcriptional activities in response to stress.* Mol Cell Biol, 2001. **21**(8): p. 2743-54.
10. Matsuoka, S., et al., *ATM and ATR substrate analysis reveals extensive protein networks responsive to DNA damage.* Science, 2007. **316**(5828): p. 1160-6.
11. Hsueh, K.W., et al., *A novel Aurora-A-mediated phosphorylation of p53 inhibits its interaction with MDM2.* Biochim Biophys Acta, 2013. **1834**(2): p. 508-15.
12. Sykes, S.M., et al., *Acetylation of the p53 DNA-binding domain regulates apoptosis induction.* Mol Cell, 2006. **24**(6): p. 841-51.
13. Bech-Otschir, D., et al., *COP9 signalosome-specific phosphorylation targets p53 to degradation by the ubiquitin system.* EMBO J, 2001. **20**(7): p. 1630-9.
14. Tang, Y., et al., *Acetylation is indispensable for p53 activation.* Cell, 2008. **133**(4): p. 612-26.
15. Wu, L., et al., *Aurora B interacts with NIR-p53, leading to p53 phosphorylation in its DNA-binding domain and subsequent functional suppression.* J Biol Chem, 2011. **286**(3): p. 2236-44.
16. Gully, C.P., et al., *Aurora B kinase phosphorylates and instigates degradation of p53.* Proc Natl Acad Sci U S A, 2012. **109**(24): p. E1513-22.

17. Liu, Q., et al., *Aurora-A abrogation of p53 DNA binding and transactivation activity by phosphorylation of serine 215.* J Biol Chem, 2004. **279**(50): p. 52175-82.

18. Wang, Y.H., et al., *Identification and characterization of a novel p300-mediated p53 acetylation site, lysine 305.* J Biol Chem, 2003. **278**(28): p. 25568-76.

19. Yang, F., et al., *Phosphoproteome profiling of human skin fibroblast cells in response to low- and high-dose irradiation.* J Proteome Res, 2006. **5**(5): p. 1252-60.

20. Abraham, J., et al., *Post-translational modification of p53 protein in response to ionizing radiation analyzed by mass spectrometry.* J Mol Biol, 2000. **295**(4): p. 853-64.

21. Sakaguchi, K., et al., *DNA damage activates p53 through a phosphorylation-acetylation cascade.* Genes Dev, 1998. **12**(18): p. 2831-41.

22. Joubel, A., et al., *Identification of new p53 acetylation sites in COS-1 cells.* Mol Cell Proteomics, 2009. **8**(6): p. 1167-73.

23. Xia, Y., et al., *Phosphorylation of p53 by IkappaB kinase 2 promotes its degradation by beta-TrCP.* Proc Natl Acad Sci U S A, 2009. **106**(8): p. 2629-34.

24. Ou, Y.H., et al., *p53 C-terminal phosphorylation by CHK1 and CHK2 participates in the regulation of DNA-damage-induced C-terminal acetylation.* Mol Biol Cell, 2005. **16**(4): p. 1684-95.

25. Huang, J., et al., *p53 is regulated by the lysine demethylase LSD1.* Nature, 2007. **449**(7158): p. 105-8.

26. Lu, H., et al., *The CDK7-cycH-p36 complex of transcription factor IIH phosphorylates p53, enhancing its sequence-specific DNA binding activity in vitro.* Mol Cell Biol, 1997. **17**(10): p. 5923-34.

27. Gu, W. and R.G. Roeder, *Activation of p53 sequence-specific DNA binding by acetylation of the p53 C-terminal domain.* Cell, 1997. **90**(4): p. 595-606.

28. Chuikov, S., et al., *Regulation of p53 activity through lysine methylation.* Nature, 2004. **432**(7015): p. 353-60.

29. Huang, J., et al., *Repression of p53 activity by Smyd2-mediated methylation.* Nature, 2006. **444**(7119): p. 629-32.

30. Huang, J., et al., *G9a and Glp methylate lysine 373 in the tumor suppressor p53.* J Biol Chem, 2010. **285**(13): p. 9636-9641.

31. Shi, X., et al., *Modulation of p53 function by SET8-mediated methylation at lysine 382.* Mol Cell, 2007. **27**(4): p. 636-46.

32. Kachirskaia, I., et al., *Role for 53BP1 Tudor domain recognition of p53 dimethylated at lysine 382 in DNA damage signaling.* J Biol Chem, 2008. **283**(50): p. 34660-6.

33. Li, A.G., et al., *An acetylation switch in p53 mediates holo-TFIID recruitment.* Mol Cell, 2007. **28**(3): p. 408-21.

34. Sakaguchi, K., et al., *Phosphorylation of serine 392 stabilizes the tetramer formation of tumor suppressor protein p53.* Biochemistry, 1997. **36**(33): p. 10117-24.
